# Vav proteins maintain epithelial traits in breast cancer cells using *miR-200c*-dependent and independent mechanisms

**DOI:** 10.1038/s41388-018-0433-7

**Published:** 2018-08-07

**Authors:** L. Francisco Lorenzo-Martín, Carmen Citterio, Mauricio Menacho-Márquez, Javier Conde, Romain M. Larive, Sonia Rodríguez-Fdez, Ramón García-Escudero, Javier Robles-Valero, Myriam Cuadrado, Isabel Fernández-Pisonero, Mercedes Dosil, María A. Sevilla, María J. Montero, Pedro M. Fernández-Salguero, Jesús M. Paramio, Xosé R. Bustelo

**Affiliations:** 1 Centro de Investigación del Cáncer, 37007 Salamanca, Spain; 2 Instituto de Biología Molecular y Celular del Cáncer, 37007 Salamanca, Spain; 3 Centro de Investigación Biomédica en Red de Cáncer (CIBERONC), CSIC-University of Salamanca, 37007 Salamanca, Spain; 4 CIEMAT, 28040 Madrid, Spain; 5 Department of Biochemistry & Molecular Biology, University of Salamanca, 37007 Salamanca, Spain; 6 Department of Physiology & Pharmacology, University of Salamanca, 37007 Salamanca, Spain; 7 Department of Biochemistry, Molecular Biology & Genetics, University of Extremadura, 06071 Badajoz, Spain; 8 Present Address: Laboratorio Max Planck de Biología Estructural, Química y Biofísica Molecular, Rosario, Argentina; 9 Present Address: Institut des Biomolécules Max Mousseron-UMR, 5247 Montpellier, France

**Keywords:** RHO signalling, Breast cancer, Transcriptomics, Non-coding RNAs

## Abstract

The bidirectional regulation of epithelial–mesenchymal transitions (EMT) is key in tumorigenesis. Rho GTPases regulate this process via canonical pathways that impinge on the stability of cell-to-cell contacts, cytoskeletal dynamics, and cell invasiveness. Here, we report that the Rho GTPase activators Vav2 and Vav3 utilize a new Rac1-dependent and *miR-200c*-dependent mechanism that maintains the epithelial state by limiting the abundance of the Zeb2 transcriptional repressor in breast cancer cells. In parallel, Vav proteins engage a *mir-200c*-independent expression prometastatic program that maintains epithelial cell traits only under 3D culture conditions. Consistent with this, the depletion of endogenous Vav proteins triggers mesenchymal features in epithelioid breast cancer cells. Conversely, the ectopic expression of an active version of Vav2 promotes mesenchymal-epithelial transitions using E-cadherin-dependent and independent mechanisms depending on the mesenchymal breast cancer cell line used. In silico analyses suggest that the negative Vav anti-EMT pathway is operative in luminal breast tumors. Gene signatures from the Vav-associated proepithelial and prometastatic programs have prognostic value in breast cancer patients.

## Introduction

The EMT is associated with the acquisition of a variety of malignant traits in cancer cells such as motility, invasiveness, metastasis, stemcellness, and chemoresistance [1–5]. This process can be regulated by multiple signaling and regulatory elements that include, among many others, the transforming growth factor β (TGFβ) pathway, protein tyrosine kinases, the Ras–Raf–ERK signaling cascade, the PI3Kα–mTOR axis, the Rho GTPase family, noncoding RNAs, and transcriptional factors such as those belonging to the Snail, Twist, and Zeb families [1, 4, 6]. These cascades impinge on the stability of cell-to-cell contacts, cytoskeletal dynamics, chemoresistance-associated metabolic routes, and stemcellness-linked transcriptional factors and noncoding RNAs [1, 4]. For example, the DNA binding transcriptional repressors of the Zeb family inhibit the expression of proteins involved in cell-to-cell contacts such as E-cadherin (Cdh1), plakophilin 2 (Pkp2), connexins (Gjb2, Gjb3), and claudin 4 (Clnd4) [4, 7]. In addition, they inhibit the expression of microRNAs associated with the regulation of stem cell-like and malignant properties of cancer cells [8].

Current evidence indicates that RhoA, the two Rac1 isoforms, and Cdc42 play both pro-EMT and anti-EMT roles in cancer cells [4]. Hence, the stimulation of RhoA can lead in some instances to increased motility via the stimulation of downstream elements such as Rho-associated protein kinases and the serum responsive factor [4, 9]. This process is mediated by the TGFβ-mediated expression of the upstream Rho guanosine nucleotide exchange factor (GEF) Net1 [10]. However, the elimination of RhoA signaling can also elicit EMT due to the dissolution of tight junctions [4]. As a result, this pathway is silenced in some EMT conditions via either the ubiquinylation-mediated proteosomal degradation of RhoA [11] or the microRNA-mediated depletion of both *RHOA* and *NET1* transcripts [10, 12]. Likewise, Rac1b and Cdc42 can promote EMT in some cases through the stimulation of Pak and reactive oxygen production in epithelial cells [13, 14]. However, the activity of the Rac1 GEF Tiam1 and the GTPase Rac1 has been also shown to contribute to maintain the epithelial state of cells by controlling the stability of E-cadherin at the plasma membrane and the localization of the ALCAM adhesive protein at cell-to-cell contacts [15–17]. Given the multipronged nature of the signaling pathways regulated by Rho family proteins [18], it is likely that other regulatory and effector mechanisms can probably participate in EMT modulation.

The three mammalian Vav proteins (Vav1, Vav2, and Vav3) are Rho GEFs directly regulated by direct tyrosine phosphorylation [19]. These proteins are involved in large variety of protein tyrosine kinase-associated physiological and pathological processes, including metabolic syndrome [20], cardiovascular disease [21–23], fibrosis [24], and cancer [19, 25–28]. In the case of breast cancer, we have recently shown that the expression of Vav2 and Vav3 is important for both the primary tumorigenesis and lung metastasis formation [26]. Interestingly, genome-wide expression profiling experiments revealed that these two proteins control a large fraction of the transcriptomal landscape of breast cancer cells using Vav2-specific, Vav3-specific, redundant, and Vav2;Vav3 synergistic pathways [26]. The latter ones are key for the Vav-dependent malignant properties of breast cancer cells [26]. As a result, the defects exhibited by *Vav2;Vav3*-deficient cancer cells in primary tumorigenesis and lung metastasis can be only rescued upon the coexpression of both Vav proteins [26].

During the course of the foregoing studies, we found that Vav2 and Vav3 proteins also control both the epithelial phenotype and chemosensitivity status of breast cancer cells. Here, we report that these new functions entail the regulation of *miR-200c*-dependent and independent gene expression programs. Unlike the foregoing Vav-dependent tumorigenic and metastatic functions, the regulation of the *miR-200c* pathway can be redundantly done by the single Vav2 and Vav3 proteins. Further underscoring the relevance of these data, we also demonstrate that the transcriptomal signatures linked to the Vav-dependent prometastatic and *miR-200c*-associated EMT programs have prognostic value for breast cancer patients.

## Results

### Vav2 and Vav3 are required to maintain epithelial traits in breast cancer cells

**Fig. 1:**
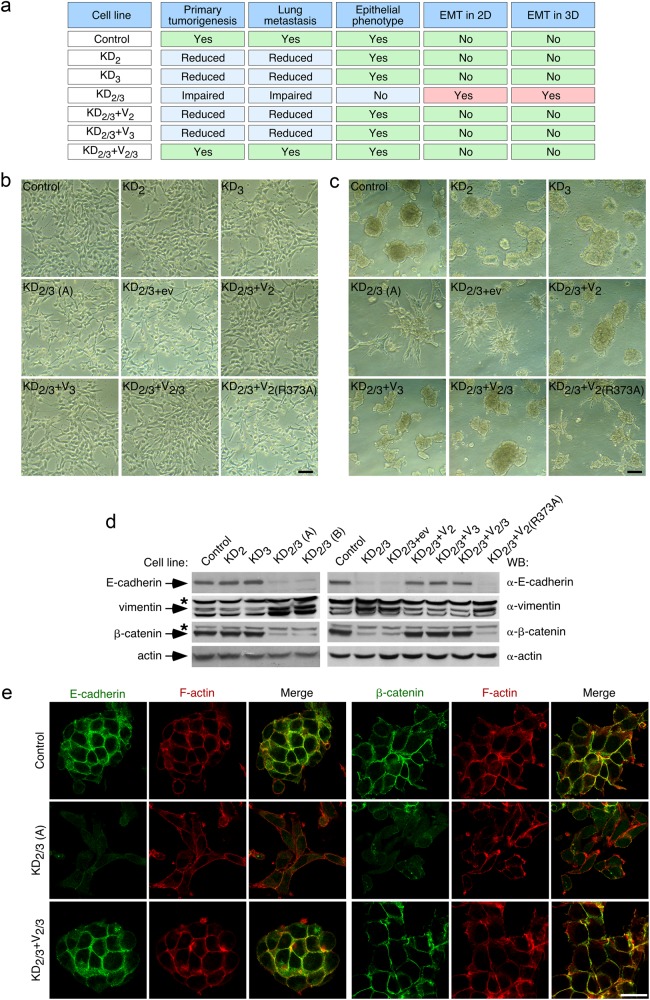
Vav2 and Vav3 are required to maintain epithelial traits in breast cancer cells. **a** Defects shown by indicated 4T1 cell lines on primary tumorigenesis and lung metastasis according to earlier work [26]. The epithelial and mesenchymal phenotypes scored in the current work are also included. **b**, **c** Representative example of the morphology of indicated 4T1 cell lines in 2D (**b**) and 3D (**c**) cultures (*n* = 3 independent experiments). Scale bars, 50 μm. KD_2/3 (A)_ and KD_2/3 (B)_ are two independent clones of Vav2;Vav3 knockdown 4T1 cells [26] (Table S1). **d** Representative immunoblot showing the abundance of indicated endogenous proteins in lysates from 4T1 cells lines shown on top (*n* = 3 independent experiments). The primary antibody used in the immunoblot is shown in the right. For loading control, we used the abundance of endogenous actin. Asterisks mark nonspecific bands. **e** Representative immunofluorescence analysis showing the abundance and subcellular localization of endogenous E-cadherin (green color), rhodamine phalloidin-stained F-actin (red color), and β-catenin (green color) in indicated 4T1 cell lines (left) (*n* = 3 independent experiments). Scale bar, 25 μm

During an earlier work aimed at characterizing the role of Vav2 and Vav3 in breast cancer [26], we generated a collection of *Vav2* (KD_2_), *Vav3* (KD_3_), and double *Vav2*;*Vav3* (KD_2/3_) knockdown 4T1 cells. In parallel, we generated “rescued” cell lines by reexpressing Vav2 (KD_2/3_+V_2_ cells), Vav3 (KD_2/3_+V_3_ cells), Vav2 plus Vav3 (KD_2/3_+V_2/3_ cells), or a catalytically inactive Vav2 version (R373A point mutant) (KD_2/3_+V_2(R373A)_ cells) in KD_2/3_ cells (Supplemental Table S1). The expected level of expression of the indicated proteins in each of those cell lines was confirmed using both Western blot and quantitative RT-PCR (qRT-PCR) analyses [26]. The effect of these genetic alterations in the primary tumorigenesis and metastatic properties of 4T1 cells was also characterized [26] (for a scheme, see Fig. 1a). The use of 4T1 cells has a number of experimental advantages, including their high metastatic potential, possibility of xenotransplant them in the mammary fat pads of immunocompetent mice, and the existence of nonmetastatic counterparts (67NR, 168FARN, 4TO7 cells) that make it possible the evaluation of gain-of-function effects of signaling routes in specific stages of the metastatic dissemination cascade [29]. These cells are also useful in our case because, similarly to human tumors, they all express both Vav2 and Vav3 [26]. This feature allows the investigation of redundant, isoform-specific, and synergistic relationships of these proteins in the malignant properties of breast cancer cells.

When analyzed in standard cell cultures, we observed in new set of experiments that the single elimination of Vav2 and Vav3 does not alter the typical epithelial morphology exhibited by the parental 4T1 cells (Fig. 1b). The KD_2_ and KD_3_ cell clones, similarly to the parental cells, also form well defined cell clusters (Fig. 1c) and tumors with an epithelial structure (Supplemental Fig. S1) when grown in 3D culture and the mammary fat pad, respectively. Despite this, we have shown before that these cells show defects in both primary tumorigenesis and lung metastasis (Fig. 1a) [26]. By contrast, we observed in these new experiments that the cells lacking the two Vav family proteins exhibit a mesenchymal phenotype in 2D culture (Fig. 1b), 3D culture (Fig. 1c), and in primary tumors (Fig. S1). Consistent with the foregoing observations, the simultaneous elimination of Vav2 and Vav3, but not of each Vav protein alone, leads to a marked reduction and elevation in the abundance of E-cadherin and vimentin, respectively (Fig. 1d). These changes are typically associated with EMT in a variety of cell lineages [1, 4, 5]. We further confirmed the loss of cell-cell contacts and E-cadherin expression in KD_2/3_ cells using immunofluorescence techniques (Fig. 1e). Additional analyses indicated that KD_2/3_ cells, but not the KD_2_ and KD_3_ counterparts, display reduced abundance of β-catenin according to both Western blot (Fig. 1d) and immunofluorescence (Fig. 1e) analyses. As a result, we could not detect the typical translocation of this protein to the nucleus usually seen upon EMT activation and E-cadherin loss (Fig. 1e). These results indicate that Vav2 and Vav3 act cooperatively in the maintenance of the epithelial morphology in 4T1 cells. In agreement with this idea, we could rescue the epithelial morphology in both 2D and 3D cultures (Fig. 1b, c) as well as parental cell-like expression levels of E-cadherin, vimentin, and β-catenin (Fig. 1d) by simply reexpressing either Vav2 or Vav3 in the KD_2/3_ cells. In fact, the coexpression of both proteins does not elicit any further additive effect in any of those experimental parameters (Fig. 1b–d). This rescue relies on the activation of catalysis-dependent routes, because the expression of the catalytically inactive Vav2^R373A^ protein does not change any of the mesenchymal features exhibited by the KD_2/3_ cells (Fig. 1b–d). These results indicate that the catalytic pathways of Vav2 and Vav3 contribute to maintain the epithelial phenotype of 4T1 cells. Unlike the case of the previously described Vav-dependent tumorigenic and metastatic properties [26], these data also indicate that this new Vav-dependent function can be redundantly executed by either Vav2 or Vav3.

### Vav2 and Vav3 regulate E-cadherin and β-catenin using different mechanisms

**Fig. 2:**
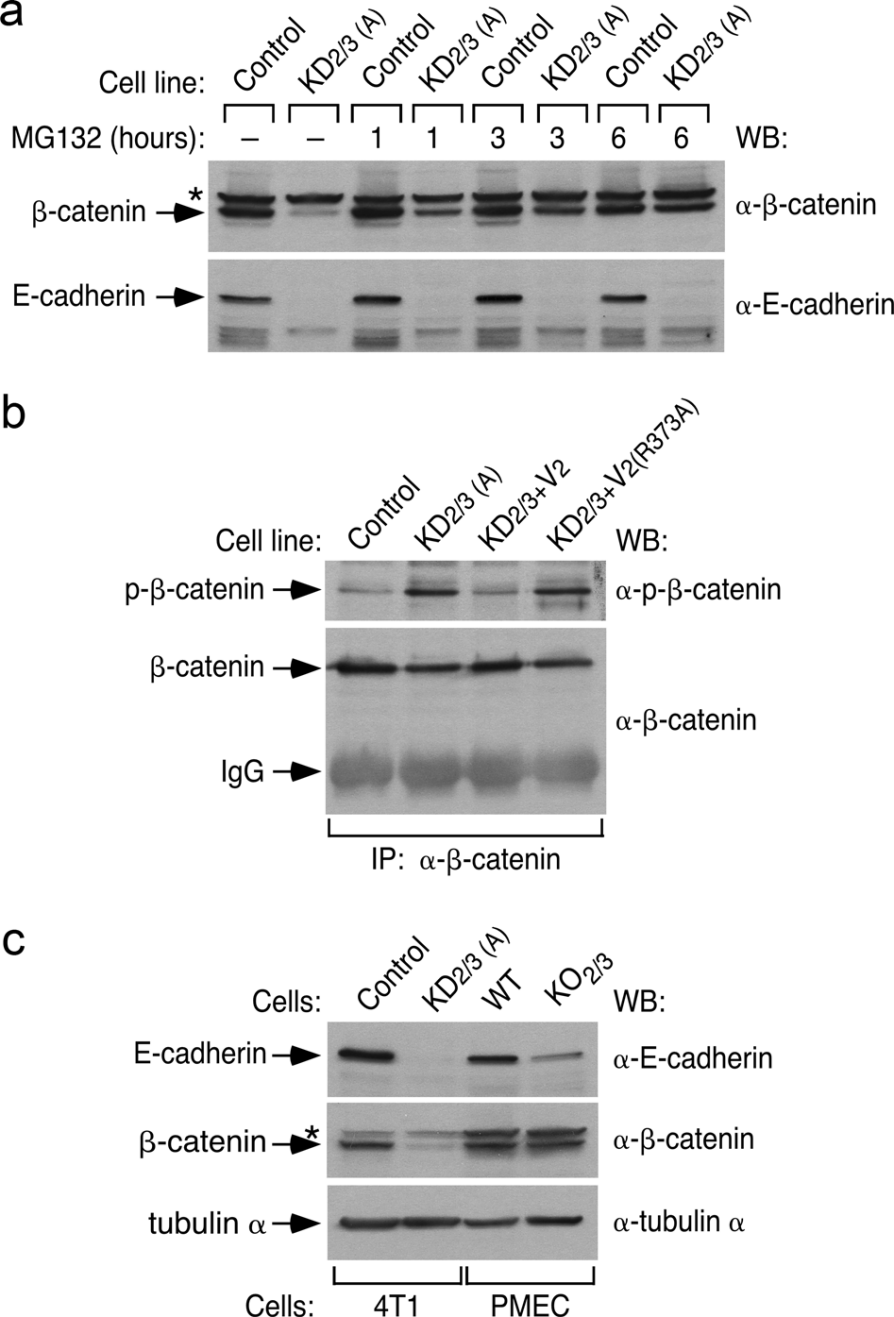
Vav2 and Vav3 regulate E-cadherin and β-catenin using different mechanisms. **a** Representative immunoblot showing the abundance of endogenous β-catenin (top panel) and E-cadherin (bottom panel) in indicated 4T1 cell lines and experimental conditions (top) (*n* = 3 independent experiments). **b** Example of the phosphorylation level (top panel) of endogenous β-catenin immunoprecipitated (IP) from indicated 4T1 cell lines (top). As control, the filter was reblotted with antibodies to total β-catenin (bottom panel) (*n* = 3 independent experiments). IgG, immunoglobulin band derived from the antibody used in the immunoprecipitation step. To ensure comparable levels of the immunoprecipitated β-catenin, we used in the case of KD_2/3_ cells 10-fold more protein extracts than in the case of control cells. **c** Abundance of endogenous E-cadherin (top panel), β-catenin (middle panel), and tubulin α (loading control, bottom panel) in indicated cell extracts (*n* = 2 independent experiments). WT and KO_2/3_, primary mammary epithelial cells (PMEC) from wild-type and *Vav2*^–/–^;*Vav3*^–/–^ mice, respectively. In **a** and **c**, asterisk label nonspecific bands

Since the steady-state abundance of β-catenin is primarily controlled by proteosomal degradation, we evaluated the effect of a proteosome inhibitor (MG132) in the levels of β-catenin and E-cadherin in control and KD_2/3_ cells. These experiments indicated that MG132 promotes the progressive restoration of β-catenin abundance in KD_2/3_ cells (Fig. 2a). As a result, the total amount of β-catenin found in control and KD_2/3_ cells reaches similar levels after six hours of incubation with this inhibitor (Fig. 2a). By contrast, MG132 does not affect the β-catenin abundance in the control cells (Fig. 2a). Consistent with the increased ratio of β-catenin degradation found in KD_2/3_ cells, we observed that this protein exhibits higher levels of phosphorylation in these cells (Fig. 2b). As in the case of EMT-related phenomena (Fig. 1b–d), normal levels of β-catenin can be restored upon the reexpression of wild-type Vav2 in KD_2/3_ cells (Fig. 2b). This does not occur when the catalytically dead Vav2^R373A^ protein is used in these rescue experiments (Fig. 2b). MG132 cannot restore normal E-cadherin levels in KD_2/3_ cells (Fig. 2a), indicating that the Vav-mediated regulation of E-cadherin and β-catenin is mechanistically different in 4T1 cells.

To assess whether the effect of Vav2 and Vav3 in the abundance of these two proteins could be generalized to primary cells, we evaluated the status of these two proteins on extracts from wild-type and *Vav2*^–/–^;*Vav3*^–/–^ primary mammary epithelial cells. We found a reduction in the abundance of E-cadherin in the latter extracts (Fig. 2c), although this defect is milder than the initially detected in the KD_2/3_ cells (Fig. 2c). By contrast, the levels of β-catenin are similar between control and *Vav2*^–/–^;*Vav3*^–/–^ cells (Fig. 2c). The amount of E-cadherin present in *Vav2*^–/–^;*Vav3*^–/–^ mice seems sufficient to maintain epithelial integrity in this case, because we found no histological or developmental defects in the mammary glands of these animals (RML and XRB, data not shown). These results suggest that the impact of Vav signaling on β-catenin abundance is cell type-specific.

### The Vav coding transcriptome and microRNAome are linked to EMT programs

We next utilized data from genome-wide mRNA and microRNA expression profiling experiments to find clues about the role of Vav proteins in the maintenance of the epithelial phenotype of 4T1 cells. Using the previously described Vav2;Vav3-dependent coding transcriptome [26], we observed by in silico GSEA (gene set enrichment analyses) that the transcriptome of KD_2/3_ shares significant similarity to those elicited under EMT conditions [30] (Fig. S2A). Consistent with this, the focalized analysis of the KD_2/3_-specific transcriptome revealed the expected downregulation (Fig. S2B) and upregulation (Fig. S2C) of mRNAs typically associated with the epithelial and mesenchymal state, respectively. Those include the *Cdh1* and *Vim* mRNAs (Fig. S2B) whose protein products were found already deregulated in our Western blot analyses (Figs. 1d and 2a,c). We also detected the upregulation of many mRNAs encoding factors commonly linked to chemoresistance, including upstream regulators, the Abcc3 drug transporter, and a large number of phase I and phase II drug metabolizing enzymes (Fig. S2D). This is probably functionally relevant, because KD_2/3_ cells exhibit more resistance than controls to the chemotherapy agents paclitaxel, doxorubicin and etoposide (Fig. S2E). This property is eliminated upon the reexpression of wild-type Vav2 in those cells (Fig. S2E). Confirming the lack of activation of the β-catenin pathway in KD_2/3_ cells, we could not find any enrichment of β-catenin-related gene signatures in these cells (LFLM and XRB, unpublished data). Further analyses of the Vav2;Vav3-dependent transcriptome revealed the upregulation of a very limited number of transcripts encoding proteins usually linked to the induction of EMT in KD_2/3_ cells [1, 4]. Those included the transcriptional factor Zeb2, two histone deacetylases (Hdac2, Hdac4), and three subunits of the transforming growth factor receptor (TGFβR1, TGFβR2, TGFβR3) (Fig. S2F). Unlike the case of Zeb2, we did not detect statistically significant variations in *Zeb1*, *Twist*, and *Snai* family mRNAs in these analyses (Fig. S2F).

**Fig. 3:**
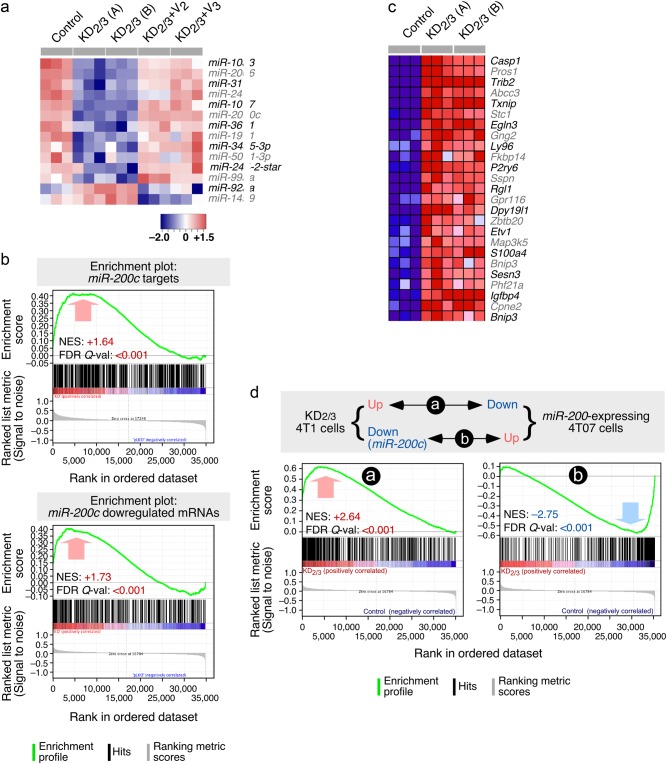
The Vav coding transcriptome and microRNAome are linked to EMT programs. **a** Heatmap of the microRNAs that are up- (red) and downregulated (blue) in indicated 4T1 cell lines (top). Triplicates for each cell line (columns) are shown. Relative changes in abundance are shown in color gradients according to the scale shown at the bottom. **b** GSEA showing the overlap of the Vav-regulated 4T1 cell transcriptome with the *miR-200c*-dependent transcriptome previously described in endometrium (top) and breast (bottom) cancer cells. The normalized enrichment scores (NES) and false discovery rate values (FDR, using *q* values) are indicated inside each GSEA graph. *Q*-val, *q* value. **c** Heatmap showing the expression of the top 25 leading-edge genes that show overlap between the *miR-200c*- and Vav2;Vav3-dependent transcriptome in indicated cell lines (top). Changes in abundance are plotted as in **a**. **d** Top panel, scheme of the similarities expected according to the mechanistic model proposed in this study. Bottom panels, GSEAs showing the similarities of the transcriptomal subsets indicated in top. The NES and FDR values are shown inside each graph as indicated in Fig. 3b. Red and blue values represent positive and negative correlation between the two analyzed transcriptomes

New microRNA Affymetrix experiments in control, KD_2/3_, and “rescued” 4T1 cells revealed that the impact of the loss of Vav proteins in the microRNAome (14 microRNAs, Fig. 3a) is lower than in the case of the coding transcriptome (2,411 mRNAs). These alterations are mainly downregulation events, since only two microRNAs (*miR-92a*, *miR-149*) display upregulated levels in the KD_2/3_ cells when compared to controls (Fig. 3a). The wild-type-like abundance of all these noncoding RNAs is fully restored when either Vav2 or Vav3 are expressed back in the KD_2/3_ cells (Fig. 3a), further indicating that both Vav proteins redundantly contribute to this regulatory step. Many of these microRNAs probably exert a wide effect in the coding transcriptome of 4T1 cells, because GSEAs indicate the existence of multiple Vav-dependent mRNAs that harbor consensus target sequences for *miR-103* (Fig. S3A), *miR-107* (Fig. S3A), *miR-206* (Fig. S3B), *miR-200c* (Fig. S3C), *miR-24* (Fig. S3D), and *miR-31* (Fig. S3E). The function of other microRNAs is uncertain (i.e., *miR-361*) according to the statistical criteria used in these analyses (Fig. S3F). Out of these microRNAs, *miR-200c* has been clearly associated with the maintenance of the epithelial state through the elimination of the pro-EMT transcriptional factor Zeb2 [31]. Given that the *Zeb2* mRNA is upregulated in Vav2;Vav3-depleted 4T1 cells (Fig. S2F), these results suggested that Vav proteins were probably involved in the expression of *miR-200c* and the concomitant repression of Zeb2 in 4T1 cells. Consistent with this hypothesis, we also observed that two microRNAs (*miR-103*, *miR-107*; Fig. 3a) and a number of coding RNAs (*Cdh1*, *Pkp2*, *Gjb3*, *Clnd4*; Fig. S2B) specifically downmodulated in the KD_2/3_ cells are known to be transcriptionally repressed by Zeb2 [7]. The importance of the downregulation of *miR-200c* is further underscored using GSEAs, as they reveal that a large percentage of the previously characterized *miR-200c*-regulated transcriptome of endometrial and breast cancer cells [32–34] becomes upregulated upon depletion of Vav2 and Vav3 in 4T1 cells (Fig. 3b,c). Likewise, GSEAs reveal high levels of inverse correlation between the Vav2;Vav3-dependent 4T1 transcriptome and the mRNA landscape induced by the ectopic expression of the entire *miR-200* cluster in mesenchymal 4TO7 breast cancer cells (which express very low amounts of both *miR-200c* and E-cadherin) [35] (Fig. 3d). A lower, although statistically significant similarity is also observed when these GSEAs are performed with the E-cadherin-regulated 4TO7 transcriptome [35] (Fig. S3G). These results suggest that the depletion of *miR-200c* has a larger impact than the loss of E-cadherin in the transcriptome of KD_2/3_ cells.

### Vav2 and Vav3 regulate the *miR-200c*–Zeb2 axis in 4T1 cells

**Fig. 4:**
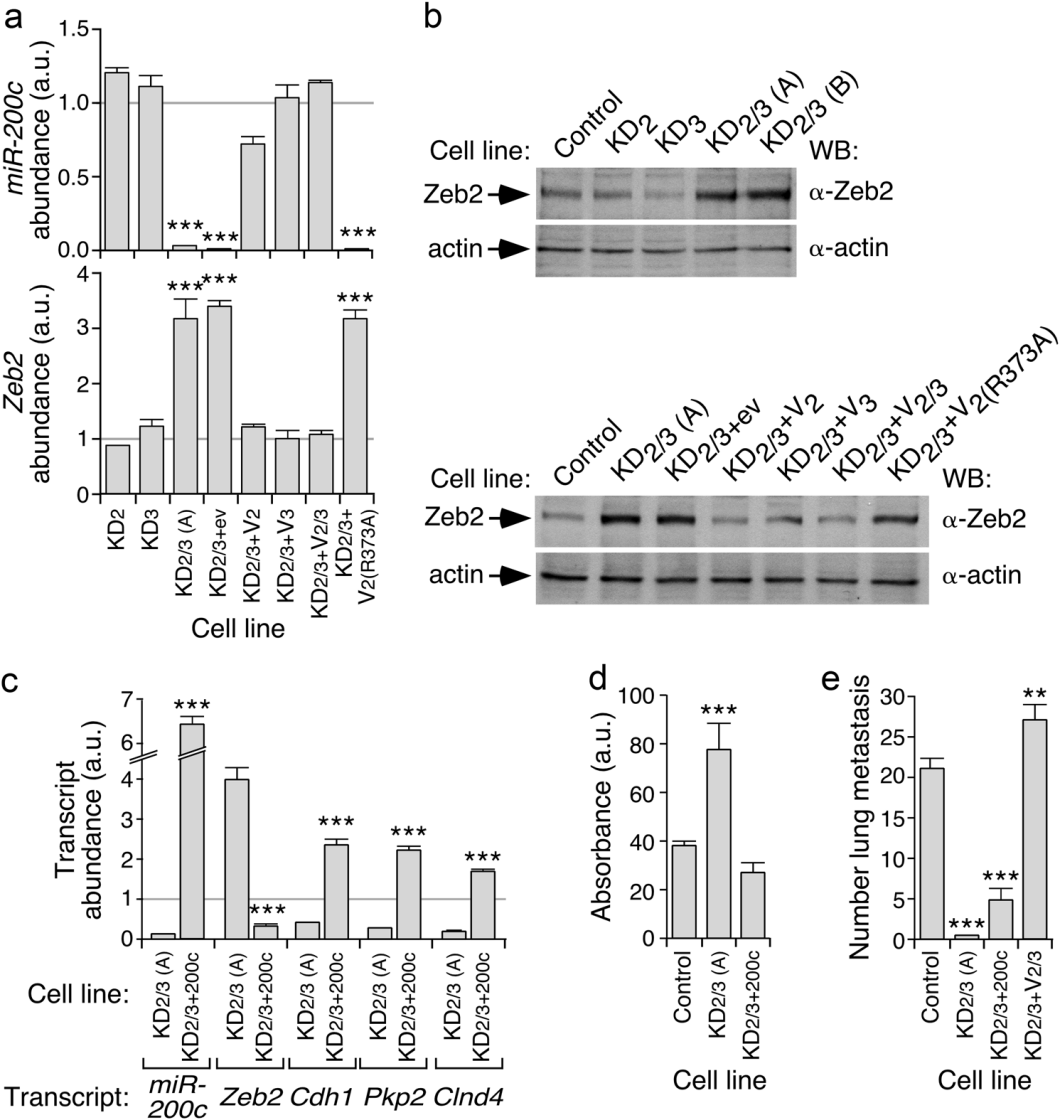
Vav2 and Vav3 regulate the *miR-200c*–Zeb2 axis in 4T1 cells. **a** qRT-PCR showing the abundance of *miR-200c* (top) and *Zeb2* mRNA (bottom) in indicated 4T1 cell lines. Expression values are plotted relative to the levels of the indicated transcript in the parental cell line (which was given an arbitrary value of 1 and represented in the figure as a gray horizontal lane). ***P ≤ 0.001 (*n* = 3 independent experiments, each performed in triplicate). a.u., arbitrary units. **b** Representative immunoblot showing the abundance of Zeb2 and actin (loading control) in indicated cell lines (top) (*n* = 3 independent experiments). **c** qRT-PCR showing the abundance of indicated *miR-200c* and specific transcripts (bottom) in indicated 4T1 cell derivatives. Expression values are plotted as in **a**. ***P ≤ 0.001 (*n* = 3 independent experiments, each performed in triplicate). **d** Response of indicated 4T1 cell derivatives (left) to doxorubicin (500 ng/ml). Values represent the variation relative to untreated cells (which was given an arbitrary value of 100). ***P ≤ 0.001 (*n* = 3 independent experiments, each performed in triplicate). **e** Metastasis formed in the lung by the indicated intravenously injected cells. **P ≤ 0.01; ***P ≤ 0.001 (*n* = 4 animals/cell line)

In agreement with the foregoing gene expression profiling data, we found using qRT-PCR experiments that the abundance of *miR-200c* and the *Zeb2* mRNA decreases and increases in KD_2/3_ cells, respectively (Fig. 4a). The elevation in the abundance of Zeb2 protein is also observed in KD_2/3_ but not in KD_2_ or KD_3_ cells (Fig. 4b), thus mimicking the results found with the evolution of epithelial/mesenchymal-associated markers in Fig. 1. Further linking the deregulation of the *miR-200c*–Zeb2 axis to the mesenchymal phenotype of KD_2/3_ cells, we observed that the abundance of the *miR-200c* (Fig. 4a), the *Zeb2* mRNA (Fig. 4a), and the Zeb2 protein (Fig. 4b) is restored to control cell-like levels upon reexpressing either wild-type Vav2 or Vav3 proteins in the KD_2/3_ cells. Such a rescue does not occur when the catalytically inactive Vav2^R373A^ proteins is ectopically expressed in those cells (Fig. 4a,b). The upregulation of the *Zeb2* mRNA and the downregulation of Zeb2 gene targets such as *Cdh1*, *Pkp2*, *Gjb3*, and *Clnd4* are both eliminated upon the forced expression of the *miR-200c* in the KD_2/3_ cells (Fig. 4c), further indicating that Vav proteins are involved in the regulation of the *miR-200c*–Zeb2–EMT axis. The expression of *miR-200c* also restores the chemosensitivity of KD_2/3_ cells to doxorubicin back to those found in control cells (Fig. 4d). However, it does not allow bypassing the metastatic defects exhibited by the KD_2/3_ cell line (Fig. 4e). This result is consistent with previous observations indicating that the coexpression of Vav2 and Vav3 is a condition sine qua non to fully restore the lung metastasis of those cells in the same experimental system [26].

### Vav proteins regulate epithelial morphology using a Rac1-dependent, PI3Kα-dependent, and Nr2f1-dependent mechanism

**Fig. 5:**
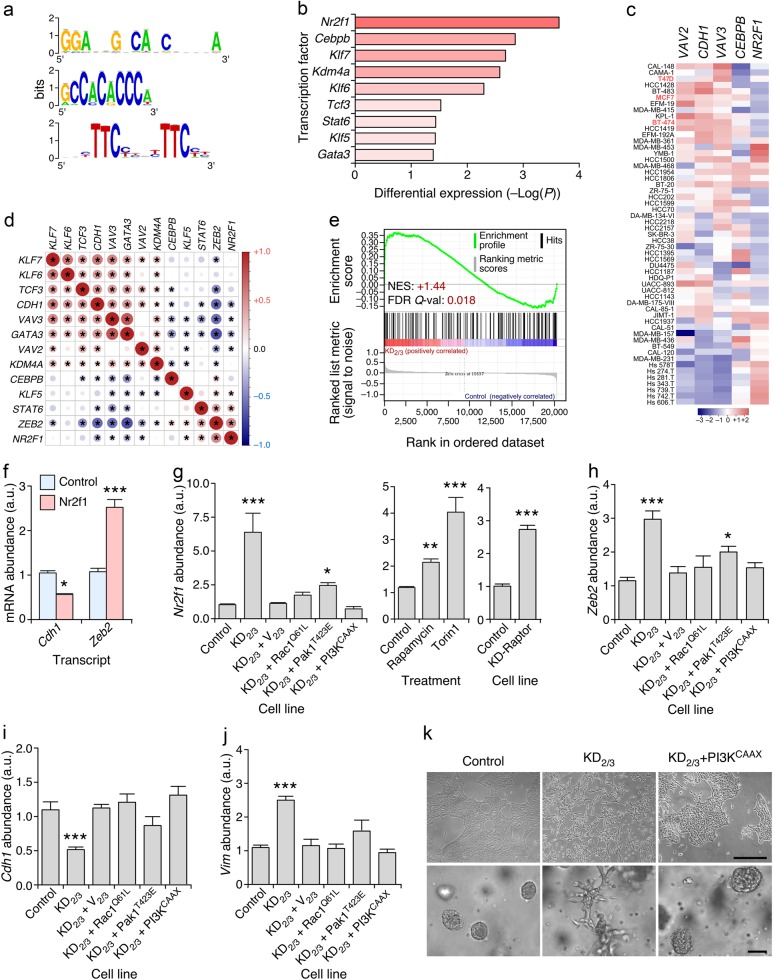
Vav proteins regulate epithelial morphology using a Rac1-dependent, PI3Kα-dependent, and Nr2f1-dependent mechanism. **a** Sequence logos of the transcription factor binding sites enriched in the promoter region of Vav-regulated microRNAs. **b** Changes in expression of the indicated transcripts in KD_2/3_ versus parental 4T1 cells according to microarray analyses [43]. **c** Abundance of indicated transcripts (top) in the Cancer Cell Line Encyclopedia collection of human breast cancer cell lines (left) [65]. In red, we show cell lines used in subsequent analyses (left). Changes in abundance are plotted as in Fig. 3a. **d** Expression correlation matrix of indicated transcripts in the human breast tumor samples present in the GSE65194 microarray dataset. Positive and negative correlation is shown in red and blue, respectively. The size and color intensity of circles are proportional to the Pearson correlation coefficient found for each transcript pair. Correlations with *P* values above the significance threshold of 0.05 have been labeled with an asterisk. **e** GSEA showing the enrichment of previously described Nr2f1 gene targets [38] in the Vav-regulated 4T1 cell transcriptome [43]. Further information about this type of analysis has been provided in the legend to Fig. 3b. **f** qRT-PCR-determined abundance of indicated transcripts (bottom) upon the transient expression of Nr2f1 in 4T1 cells. Expression values are plotted relative to the levels of the indicated transcript in the parental cell line (which was given an arbitrary value of 1). *P ≤ 0.05; ***P ≤ 0.001 (*n* = 3 independent experiments, each performed in triplicate). **g** Abundance of the *Nr2f1* mRNA in indicated 4T1 cells and experimental conditions. Experiments were performed as in **f**. *P ≤ 0.05; ***P ≤ 0.01; ***P ≤ 0.001 (*n* = 3 independent experiments, each performed in triplicate). **h**–**j** Abundance of *Zeb2* (**h**), *Cdh1* (**i**), and *Vim* (**j**) transcripts in control and indicated 4T1 cell derivatives (bottom). Experiments were performed as in **f**. ***P ≤ 0.001 (*n* = 3 independent experiments, each performed in triplicate). **k** Representative example of the morphology of indicated 4T1 cell lines in 2D (top) and 3D (bottom) cultures (*n* = 3 independent experiments). Scale bars, 50 μm

We next performed in silico and signaling studies to identify the mechanism involved in the regulation of this new Vav-dependent pathway in breast cancer cells. Bioinformatics analyses of the promoter regions of the Vav-regulated microRNAs indicated the presence of common DNA motifs (Fig. 5a) that could potentially act as target sites for transcriptional factors that are upregulated at different levels in the KD_2/3_ cells (Fig. 5b). Out of those transcriptional factors we decided to focus on Nr2f1 (also known as COUP-TFI), an orphan nuclear receptor belonging to the steroid/thyroid hormone receptor superfamily previously involved in breast cancer cell dormancy [36, 37]. This was the most likely candidate to engage this *miR-200c*–Zeb-dependent program for a number of reasons: (i) Higher levels of expression of its transcript in KD_2/3_ cells relative to the other possible candidates (Fig. 5b). (ii) A mirror-image expression of the *NR2F1* transcript with those encoding the Vav2, Vav3 and E-cadherin proteins in a large variety of human breast cancer cell lines (Fig. 5c). (iii) The inverse correlation found between the abundance of the *NR2F1* mRNA and the transcripts for other signaling elements of the pathway under analysis in this work (*VAV2*, *VAV3*, and *CDH1*) in human tumor samples according to in silico coexpression matrix analyses (Fig. 5d). (iv) The direct correlation observed between the abundance of the *NR2F1* and *ZEB2* mRNAs in human tumor samples when using coexpression matrix analyses (Fig. 5d). (v) The statistically significant enrichment of previously described Nr2f1 target genes [38] in the transcriptome of KD_2/3_ cells according to GSEAs (Fig. 5e). In agreement with this hypothesis, we observed using transient transfection experiments that the ectopic expression of Nr2f1 leads to the repression and upregulation of *Cdh1* and *Zeb2* transcripts in 4T1 cells, respectively (Fig. 5f). Furthermore, we found using qRT-PCR that the endogenous *Nrf2l* transcript is upregulated in KD_2/3_ cells when compared to control cells (Fig. 5g). This change in expression is intrinsically associated with the function of Vav proteins, because normal expression levels of the *Nr2f1* mRNA are restored in the KD_2/3_+V_2/3_ rescued cells (Fig. 5g). To get further insights on the regulation of Nr2f1 by Vav proteins, we analyzed the expression of its transcript in KD_2/3_ cells that were reconstituted with active versions of either the Rac1 GTPase (Q61L mutant) or the Pak1 serine/threonine kinase (T423E mutant). Given that the expression of *miR-200c* is also highly dependent on PI3K–AKT signaling [39], we included in these analyses a KD_2/3_ cell line stably expressing a membrane-anchored version of PI3Kα (PI3K^CAAX^). The expression of any of those three proteins resulted in the restoration of parental cell-like levels of the *Nr2f1* mRNA in the KD_2/3_ cells (Fig. 5g, left panel). These three constitutively active proteins also restored normal levels of expression of *Zeb2* (Fig. 5h), *Cdh1* (Fig. 5i) *and* Vim (Fig. 5j) transcripts in those cells. The connection of PI3Kα with Nr2f1 is probably mediated via the stimulation of the mTOR complex, since the inhibition of endogenous mTOR complexes with rapamycin and to a larger extent the torin1 inhibitor leads to the upregulation of the *Nr2f1* mRNA in the parental 4T1 cells (Fig. 5g, middle panel). Similar results are obtained when Raptor, an integral component of mTORC1 [40], is depleted in 4T1 cells (Fig. 5g, right panel). Consistent with these data, we observed that the ectopic expression of PI3K^CAAX^ restores the epithelial morphology in the KD_2/3_ cells (Fig. 5k). These results suggest that the maintenance of the epithelial phenotype by Vav proteins is mediated by a Rac1–Pak1 and PI3K–AKT–mTOR dependent mechanism that help maintaining the basal levels of E-cadherin expression in 4T1 cells.

The in silico analysis of microarray datasets also suggested that the abundance of *VAV2* and *VAV3* transcripts is associated with low and high levels of *CDH1* and *NR2F1* transcripts in human breast cancer cell lines, respectively (Fig. 5c). In line with this, we found that the knockdown of *VAV2* and *VAV3* transcripts in the epithelial T47D cell line (Fig. S4A) leads to the downregulation and upregulation of *CDH1* and *NR2F1* mRNAs, respectively (Fig. S4B). The fold change obtained in both cases was smaller than that found in 4T1 cells (compare Fig. 5 and S4B), probably because the shRNA-mediated knockdown of *VAV* family transcripts was less efficient in the human than in the mouse cell line. We also observed that the transient expression of Nr2f1 results in the reduction of *CDH1* mRNA levels in three independent human breast cancer cell lines that display an epithelial phenotype (T47D, MCF7, BT474) (Fig. S4C). Conversely, it leads to the upregulation of the *ZEB2* mRNA (Fig. S4C). Nr2f1 also reduces the abundance of E-cadherin and, to a lesser extent β-catenin proteins when ectopically expressed in the T47D cells (Fig. S4D). These results indicate that the Vav2;Vav3–Nr2f1–*miR-200c*–Zeb2–Cdh1 axis is probably conserved in both mouse and human breast cancer cell lines.

### Vav proteins regulate 3D EMT using both Zeb2- and E-cadherin-independent mechanisms

**Fig. 6:**
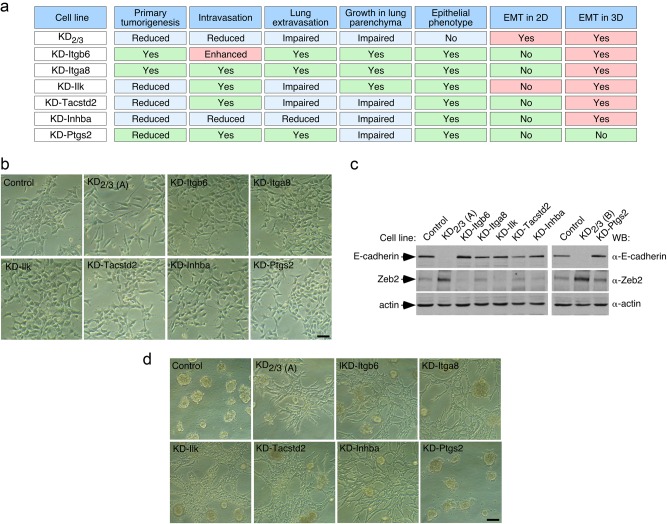
Vav proteins regulate 3D EMT using Zeb2- and E-cadherin-independent mechanisms. **a** Defects shown by the indicated 4T1 cell derivatives in primary tumorigenesis and specific steps of the metastatic dissemination to the lung [26]. The epithelial and mesenchymal status in 2D and 3D obtained in this work are also included. **b** Representative example of the morphology of indicated 4T1 cell lines in 2D culture (*n* = 3 independent experiments). Scale bar, 50 μm. **c** Representative immunoblot showing the abundance of indicated endogenous proteins in lysates from 4T1 cell line derivatives shown on top. Actin has been used as loading control (*n* = 3 independent experiments). **d** Representative example of the morphology of indicated 4T1 cell lines in 3D culture (*n* = 3 independent experiments). Scale bar, 50 μm

We have previously identified a number of Vav2;Vav3-dependent distal transcriptional targets in breast cancer cells [26]. We have shown before that the knockdown of some of those transcripts (*Itgb6*, *Itga8*) does not have any overt effect in the malignant properties of 4T1 cells, with the exception of the increased intravasation found in integrin α_8_-depleted cells [26] (for a summary, see Fig. 6a). However, we also showed in this previous study that the shRNA-mediated depletion of the mRNAs for other identified targets does negatively impact on the primary tumorigenesis (*Ilk*, *Tacstd2*, *Inhba*, *Ptgs2*), intravasation (*Inhba*), lung extravasation (*Ilk*, *Tacstd2*, *Inhba*), and/or growth in the lung niche (*Tacstd2*, *Inhba*, *Ptgs2*) [26] (Fig. 6a). To assess the role of these distal downstream elements in EMT, we decided to evaluate in the current work the morphology of knockdown cells lacking each of those mRNAs (Table S1) in both 2D and 3D cultures. Unlike the case of KD_2/3_ cells, we found no consistent alterations in the epithelial morphology (Fig. 6b), the shutdown of E-cadherin expression (Fig. 6c) or the upregulation of Zeb2 protein levels (Fig. 6c) in any of the interrogated knockdown cells in 2D cultures. This indicates that these distal transcriptional targets of the Vav pathway do not participate in the regulation of the *miR-200c*–Zeb2 axis in 4T1 cells. However, in 3D cultures, the single elimination of integrin β_6_, integrin α_8_, Ilk, Tacstd2 or inhibin βA promotes a mesenchymal-like behavior characterized by the formation of long chains of cells that maintain polarized cell-to-cell contacts and that, therefore, do not break away from their neighbors (Fig. 6d). This phenotype, which is even more extreme than that observed in the case of KD_2/3_ cells, resembles cases of partial EMT previously found in other 3D models (for a review, see Ref. [41]). By contrast, this phenotype is not observed in *Ptgs2*-depleted 4T1 cells (Fig. 6d). These results suggest that Vav proteins can utilize independent mechanisms to maintain the epithelial phenotype in 2D (the Nr2f1–*miR-200c*–Zeb2–E-cadherin axis) and 3D (the Nr2f1–*miR-200c*–Zeb2–E-cadherin axis and distal elements of the Vav-dependent transcriptome) conditions. Given that KD-Itgb6 and KD-Itga8 cells do not show any significant defect in primary tumorigenesis or metastasis (Fig. 6a), these results also indicate that the induction of this EMT-like process under 3D culture conditions does not favor per se the metastatic potential of these cells. This is consistent with the observation that KD-Ptgs2 cells are metastasis deficient despite their wild-type-like behavior in both 2D and 3D cultures (Fig. 6a).

### Constitutively active Vav2 triggers mesenchymal-epithelial transitions in mesenchymal breast cancer cells using E-cadherin dependent and independent mechanisms

We next addressed the effect of overexpressing the wild-type and constitutively active versions of Vav2 (Y172F mutant) in the morphology of the mesenchymal 67NR, 168FARN, and 4TO7 cells (Table S1). Each of these cell lines exhibit defects in specific stages of the dissemination metastatic cascade (Fig. S5A) [29]. The ectopic expression of wild-type Vav2 does not change the mesenchymal phenotype exhibited by 67NR and 168FARN cells in 2D (Fig. S5B) and 3D (Fig. S5B) cultures. It does not alter either the mesenchymal features of 4TO7 in 2D culture (Fig. S5B), although it does favor the acquisition of a more epithelial, noninvasive condition of these cells in 3D culture conditions (Fig. S5B). By contrast, we observed that the expression of Vav2^Y172F^ leads to the acquisition of an epithelial-like morphology in the three cell lines interrogated in 2D cultures (Fig. S5B). This effect is further enhanced in 3D cultures, since all Vav2^Y172F^-expressing cells form smooth spheroids under these conditions (Fig. S5B). Despite the similarity in the phenotypes observed, we observed that the mesenchymal-epithelial transition (MET) induced by Vav2^Y172F^ is mechanistically different depending upon the cell line analyzed. Thus, in good agreement with the pathway previously dissected using the 4T1 cell model, we found that the ectopic expression of Vav2^Y172F^ promotes the expected upregulation and the dowmodulation of *miR-200c* and the *Zeb2* transcript in 168FARN cells, respectively (Fig. S5C, top panel). This effect is also associated with the expected upregulation and downregulation of epithelial- (*Cdh1*, *Pkp2*, *Gjb3*) and mesenchymal-associated (*Vim*, *Fn1*, *Cdh2*) transcripts, respectively (Fig. S5C, top panel; Fig. S5D). The upregulation of the *Itgb6* and *Itgb8* transcripts is also observed upon the ectopic expression of Vav2^Y172F^ in these cells (Fig. S5C, top panel). Using Western blot analyses, we found that the ectopic expression of Vav2^Y172F^ promotes the expected increase in the abundance of both E-cadherin (Fig. S5E, top panel) and β-catenin (Fig. S5E, second panel from top) in 168FARN cells. Vav2^Y172F^ also induces an elevation in the basal levels of phospho-Akt in those cells (Fig. S5E, third panel from top). By contrast, we observed that the expression of Vav2^Y172F^ in both 67NR and 4TO7 cells does not involve a canonical MET. Hence, we did observe the expected regulation of *miR-200c*, the *Zeb2* mRNA, and the transcripts associated with the mesenchymal phenotype (*Vim*, *Fn1*, *Cdh2*) when Vav2^Y172F^ is expressed in these two cell lines (Fig. S5C, middle and bottom panels, respectively; Fig. S5D). However, the transcripts for epithelial markers become unexpectedly downregulated rather than upregulated under the same experimental conditions in those two cell lines (Fig. S5C, middle and bottom panels, respectively; Fig. S5D). Unlike the case of 168FARN cells (Fig. S5C, top panel), the effect of the ectopic expression of Vav2^Y172F^ on the abundance of the *Itgb6* and *Itgb8* transcripts is highly variable depending on the cell line analyzed (Fig. S5D, middle and bottom panels). These results indicate that the Vav-dependent pathways that contribute to the maintenance of the epithelial phenotype are probably conserved in both 4T1 and 168FARN cells. They also indicate that Vav proteins can trigger MET using hitherto unknown E-cadherin-independent mechanisms.

### *VAV* family and *ZEB2* mRNA levels are inversely correlated in human breast tumors

**Fig. 7:**
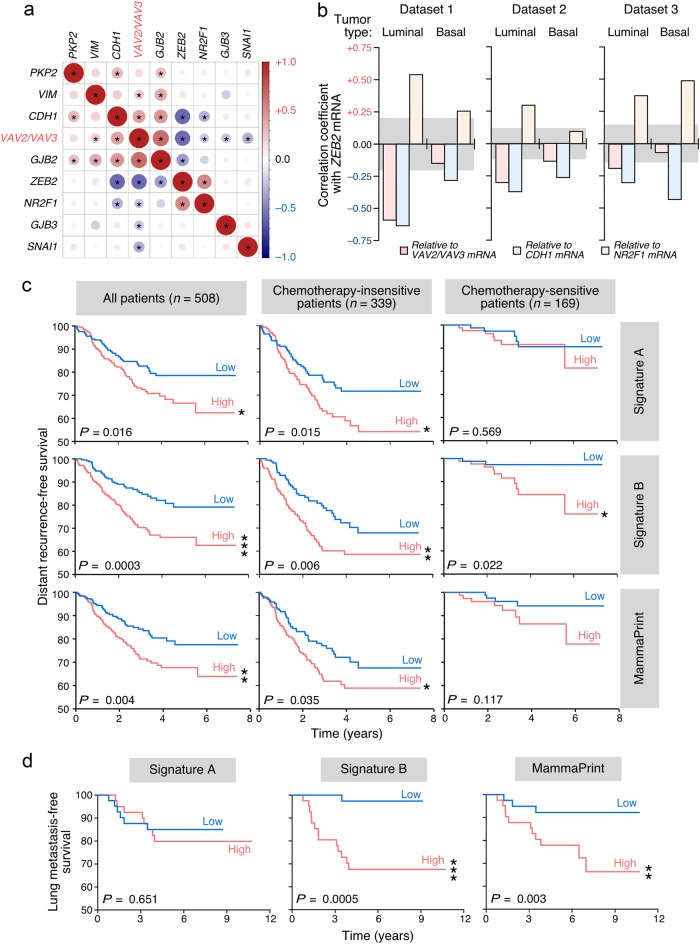
Prognostic value of Vav EMT- and lung metastasis-associated gene signatures. **a** Expression correlation matrix of indicated transcripts in human luminal breast tumor samples present in the GSE65194 microarray dataset (luminal tumors) [66]. In these analyses, the abundance of *VAV2* and *VAV3* mRNAs (*VAV*, red lettering) was pooled together to calculate the expression correlation values. The graphic representation was made as indicated in Fig. 5d. **b** Graph representing the Pearson correlation coefficient of the *ZEB2* mRNA with indicated transcripts, tumor subtypes, and microarray datasets 1 (GSE65194) [66], 2 (GSE25066) [67, 68] and 3 (GSE78958) [69]. The gray areas depict the nonstatistically significant values according to statistical threshold used in the analyses (*P* ≤ 0.05). **c**, **d** Patients were stratified into high (red lanes) and low (blue lanes) groups according to their similarity to the *miR-200c*–Vav2;Vav3-associated EMT (**c**, top panels; **d**, left panel), the Vav2;Vav3-dependent prometastatic (**c** and **d**, middle panels), and the MammaPrint (**c**, bottom panels; **d**, right panel) signatures and curves plotted according to either distant recurrence-free survival (**c**) in the indicated patient groups (top, gray boxes) or lung metastasis-free survival (**d**). Datasets used in these analyses were GSE25066 (for the survival analyses) [67, 68], GSE2603 (for the survival analyses) [70], and GSE2603 (metastasis free-survival) [70]

To assess whether the specific correlation of the expression of Vav, Nr2F1 and Zeb2 proteins found in 4T1 cells could be conserved in human tumors, we performed further coexpression matrix analyses using a large microarray dataset that contained samples from normal tissue as well as HER2^+^, luminal A, luminal B, and basal breast tumors (*n* = 1097 samples). To further strengthen these analyses, we also included in the study the correlation of the foregoing mRNAs with transcripts known to be repressed by Zeb2 and/or deregulated during EMT processes (*CDH1*, *PKP2*, *GJB2*, *GJB3*, *VIM*). This approach revealed that the combined abundance of *VAV* family *(VAV2+VAV3)* transcripts does show an inverse correlation with the expression levels of both *NR2F1* (Fig. 7a) and *ZEB2* (Fig. 7a,b, red bars) mRNAs in luminal breast tumors. It also shows the expected direct correlation with the amount of both *CDH1* and *GJB2* mRNAs present in luminal samples (Fig. 7a). Similar correlations are obtained when the expression levels of the single *VAV2* and *VAV3* mRNAs are independently used in these analyses (Fig. S6A). All those correlations are lost in the case of basal breast tumors (Fig. S6B). As control, these studies detected an inverse and a direct correlation between the expression of the *ZEB2* transcript and the amount of *CDH1* (Fig. 7a,b, blue bars) and *NR2F1* (Fig. 7a,b, brown bars) mRNAs, respectively. These results are in agreement with the known repression of CDH1 by Zeb2 [4, 7] (this work) and, in addition, with our present results showing a connection between the expression of Nr2f1 and the upregulation of Zeb2 (Figs. 4 and 5). The direct correlation between *ZEB2* and *NR2F1* transcripts is recurrently detected in the luminal tumor samples from all the microarray datasets interrogated in this study (Fig. 7a,b) whereas it shows a less consistent pattern in the case of samples from basal subtype tumors (Fig. 7b, brown bars). By contrast, the inverse correlation observed between the *ZEB2* and *CDH1* mRNAs is maintained in all the datasets surveyed regardless of the tumor subtype involved (Fig. 7b, blue bars). This suggest that the regulation of Zeb2 expression is probably subjected to different mechanisms in these two tumor subtypes. We found no statistically significant correlations of *VAV*, *NR2F1* or *ZEB2* transcripts with other putative Zeb2-regulated (*GJB3*, *PKP2*) and EMT-associated (*VIM*) transcripts (Fig. 7a and S6), suggesting that the expression of these latter mRNAs is regulated by alternative mechanisms in primary tumors. Consistent with this idea we did not find, for example, the expected inverse correlation between the abundance of *CDH1* and *VIM* mRNAs (Fig. 7a and S6). Interestingly, no statistically significant correlation was observed when all these EMT-linked mRNAs were tested against the *SNAI1* mRNA in luminal tumor-derived samples (Fig. 7a and S6A). These results suggest that the regulation of the anti-EMT program by the Vav family is mainly operative in luminal tumors. We have shown before that this cancer subtype is usually associated with increased abundance of *VAV3* transcripts [26].

### Vav EMT- and metastasis-associated gene signatures have prognostic value

We finally investigated the prognostic value of gene signatures associated with the Vav-dependent EMT and lung metastatic programs. To this end, we generated a minimal gene signature composed of 109 probe sets (90 genes) that are highly sensitive to variations in Vav pathway activity and *miR-200c* levels in breast cancer cells according to GSEAs (Fig. 3b,c; Table S2). These genes, which will be referred to hereafter as “Signature A”, are therefore directly related to the acquisition of a mesenchymal state by 4T1 cells. However, it is unrelated to the capacity of forming lung metastasis by those cells. On the other hand, we used the previously described 120 probesets (102 genes) signature (referred to hereafter as “Signature B”) that encompasses a subset of Vav2;Vav3-dependent genes that are directly correlated with the capacity of 4T1 cells to form lung metastases [26]. As comparative control, we utilized the MammaPrint gene signature currently used in the clinic [42]. These signatures were tested in microarray-derived datasets of breast cancer patients to assess the ability to stratify patients according to overall patient survival, chemoresistance-related disease outcome, and lung metastasis-free survival. We found that the prometastatic Signature B is clearly the most robust, since it can stratify patients according to all the clinical parameters chosen in these analyses (Fig. 7c,d). In fact, its prognostic power is even better than the MammaPrint signature in terms of overall survival (*P* = 0.0003 vs. 0.004), disease outcome in chemotherapy-insensitive (*P* = 0.006 vs. 0.035) and sensitive (*P* = 0.002 vs. no stratification power) patients, and in lung metastasis-free survival (*P* = 0.0005 vs. 0.003) (Fig. 7c,d). This is probably due to the functional link of this Vav2;Vav3-dependent signature with the tumorigenic and lung-associated metastasis properties of breast cancer cells (Figs. 1a and 6a) [26]. Signature A can only stratify both the global and the chemotherapy-insensitive patient cohorts according to distant recurrence-free survival (Fig. 7c). In the latter case, its prognosis value is approximately two-fold higher than the MammaPrint’s (*P* = 0.015 vs. 0.035). Interestingly, these results are consistent with the correlation of this signature with the chemoresistance status rather than with the metastatic efficiency of 4T1 cells.

## Discussion

We have reported here that Vav2 and Vav3 contribute to maintain the epithelial phenotype and associated molecular traits of breast cancer cells. This new function is mediated by two mechanisms that are different both from the mechanistic and functional point of view. On the one hand, Vav proteins mediate the basal expression of the *miR-200c* that helps maintaining the epithelial phenotype under both 2D and 3D culture conditions via the inhibition of Zeb2 expression. This pathway can be redundantly engaged by Vav2 and Vav3, requires the catalytic activity of Vav proteins and the engagement of Rac1 GTPase signaling elements such as Pak1 and PI3K. This route eventually leads to the repression of Nr2f1 expression, elevated levels of *miR-200c*, low basal levels of Zeb2, and E-cadherin levels compatible with the maintenance of the epithelial state. This pathway is linked to the maintenance of the responsiveness to chemotherapy drugs and the epithelial phenotype independently of the culture conditions used. However, it is not self-sufficient to favor the Vav-dependent prometastatic functions of 4T1 cells as inferred from the fact that KD_2/3_+V_2_ and KD_2/3_+V_3_ cells are not metastatic [43] despite the rescue of a parental cell-like epithelial phenotype (this work). On the other hand, we have shown that Vav proteins can also promote an E-cadherin-independent gene expression program that favors the epithelial state of breast cancer cells specifically under 3D conditions. This 3D-specific proepithelial pathway includes protein products derived from genes of the previously described “Vav3-specific” (integrin β_6_), “Vav family redundant” (integrin α_8_, Tacstd2), and “Vav2;Vav3 synergistic” (Ilk, inhibin βA) transcriptomal programs [26]. Unlike the case of the *mir-200c*–Zeb2 pathway, we have shown before that this “prometastatic” program is engaged using both Vav catalysis-dependent and independent mechanisms [26]. The functional segregation of these two programs is further underscored by the prognosis power exhibited by the gene signatures derived from each of them. Thus, whereas the *miR-200c*-associated signature exhibits good stratification power only in the case of the distant disease-free evolution of chemoresistant patients, the “prometastatic” gene signature does so in all clinical parameters including, in good agreement with the foregoing data, the lung metastasis-free survival of breast cancer patients. Collectively, our results indicate that Vav proteins contribute to maintain the epithelial phenotype of breast cancer cells in a signaling autonomous manner. Consistent with this view, we have also observed that the expression of constitutively active Vav2 can promote epithelial features when expressed in the otherwise mesenchymal 168FARN cell line using a similar signaling pathway. Interestingly, we have also observed that constitutively active Vav2 can promote epithelial features in the mesenchymal 67NR and 4TO7 breast cancer cells in the absence of reexpression of the *Cdh1* mRNA and other epithelial phenotype-associated transcripts. This suggests that Vav proteins can probably stimulate alternative proepithelial programs that remain to be discovered as yet. MET in the absence of E-cadherin expression has been found before by others [44]. In contrast to the present results, it has been previously reported that the third Vav family member, Vav1, drives EMT in both ovarian and pancreatic cancer cells [45, 46]. This action is mediated by the stimulation of the transcriptional factors Snail1 and Slug at least in the case of ovarian cells [46]. Whether these opposite roles reflect the existence of either isoform-specific or cell type-specific signaling programs of Vav family proteins remains to be determined.

Our current data also indicate that the regulation of the Vav-dependent transcriptomal landscape is probably more complex than previously anticipated. For example, they suggest that a significant fraction of transcripts found repressed in the Vav “redundant” transcriptome is probably regulated by microRNA-mediated degradation rather than by direct gene repression events. As a token, our analyses indicate that the changes in *miR-200c* expression can be solely responsible for ≈50% of the Vav “redundant” transcriptome found in the in Vav-deficient KD_2/3_ cells. The influence of microRNAs in the orchestration of this transcriptome can be even larger considering the multiple interconnections and feedback loops existing between microRNAs, transcriptional factors, and signaling pathways in cells [47]. It would be interesting to investigate in the near future the overall impact of the identified microRNAs in the Vav-dependent transcriptome, the specific roles they regulate in breast cancer cells, and whether Vav proteins control the expression of additional noncoding RNA species.

Current evidence indicates that Vav proteins influence a wide spectrum of biological responses in cancer cells that are associated with either primary tumorigenesis, metastasis, anti-EMT and other functions. Thus, in addition to previous [43] and current data (this work), we have recently observed that Vav proteins can influence the expression of a vast transcriptional program associated with the mevalonate pathway and its biosynthetic branches. This pathway seems to be Rac1- and PI3K–AKT–mTOR-dependent (JC and XRB, manuscript in preparation). This suggest that, if properly targeted, the inhibition of the Vav-regulated signaling route can be of potential interest for the treatment of luminal breast tumors.

To date, most of the attention in the Rho field has focused on the actions of microRNAs on the Rho GTPases, regulators, and effectors. This has led to the identification of a large number of noncoding RNAs involved in these processes (some examples are reviewed in Ref. [48]). The microRNA-mediated regulation of Vav family proteins also has been found in some sporadic cases [19, 49–52]. However, to our knowledge, no information is available on the participation of microRNAs as Rho GTPase distal downstream elements. Our results highlight the importance of identifying this “dark matter” in the field to better understand the full regulatory programs of these GTPases in physiological and pathological processes.

## Materials and methods

### Cell lines and primary cells

4T1 cells and most of their derivative cell lines used in this work (Table S1) have been described before [26, 53]. In the case of KD_2/3_+200c cells, we transduced the previously described KD_2/3 (A)_ 4T1 cells [26] with a lentiviral vector expressing *miR-200c* and GFP. Seventy-two hours later, cells were trypsinized and those expressing high levels of GFP purified by flow cytometry. DK-Raptor 4T1 cells (Table S1) were generated using a shRNA targeting the mouse *Rptor* transcript (TRCN0000077469, Sigma). Human breast cancer cell lines were generously provided by J. Arribas (Vall d’Hebron Institute of Oncology, Barcelona, Spain). T47D-KD_2_, T47D-KD_3_, and T47D-KD_2/3_ cells (Table S1) were generated using the appropriate shRNAs for human *VAV2* (TRCN0000048227, Sigma) and *VAV3* (TRCN0000047701, Sigma) transcripts. 67NR, 168FARN and 4TO7 cells have been previously described. When required, stable cell pools expressing either wild-type Vav2 (V_2_) or Vav2Y172F (V_2_(Y172)) were generated using transductions with lentiviral particles as described before [26, 53] (Table S1). In all cases, we used “control” cells that were selected upon infection with the empty lentivirus. For generation of lentiviral particles and cell transductions, see Citterio et al. [26]. Isolation of primary mammary epithelial cells from female wild-type and *Vav2*^*–/–*^;*Vav3*^–/–^ knockout animals (C57BL/10 genetic background) [22] was carried out as described [26, 54].

The culture of cells in 2D conditions was done as previously described by us [43]. 3D cultures were performed as described [55]. Briefly, eight‐well Lab Tek II chamber slides (Nalge Nunc International) were coated with 50 μl/well of ice‐cold Matrigel (BD Biosciences). Suspended cells (1.2 × 10^4^ cells in 100 μl of growth medium) were placed on top of the Matrigel and allowed to attach for 30 min at 37 °C. One hundred and fifty microliter of growth medium, containing 10% v/v of Matrigel, was added on top of cell layer and cells were then cultured at 37 °C for 4 days. Brightfield images of cell colonies were subsequently acquired and analyzed.

When required, cells were incubated with the indicated concentration of chemotherapy compounds (Cat. No. CAS 33069-62-4, CAS 25316-40-9, CAS 33419-42-0; Calbiochem-Merck Millipore). Twenty-four hours later, cells were collected, lysed in RIPA buffer (150 mM NaCl, 50 mM Tris, pH [8.0], 1.0% IGEPAL^®^ CA-630, 0.5% sodium deoxycholate, 0.1% SDS), and protein concentration measured using a Bradford assay (BioRad). Cells were alternatively treated with the mTOR inhibitors rapamycin (25 nM, Cat. No. S1039, Selleckchem) and torin1 (250 nM, Cat. No. S2827, Selleckchem) for 24 h. Cells were then collected and total RNA extracted as indicated [26].

### Immunofluorescence

Techniques and reagents were those previously described [26]. New antibodies used included E-cadherin (Cat. No. 610181, BD Biosciences) and β-catenin (Cat. No. 610153, BD Biosciences).

### Western blotting and immunoprecipitation analyses

To evaluate the abundance of endogenous proteins, 60 μg of lysates from the indicated cells were subjected to immunoblot analyses using antibodies to E-cadherin, vimentin (ab8978, Abcam), β-catenin, actin (Cat. No. A4700, Sigma), Zeb2 (Cat. No. sc-48789, Santa Cruz Biotechnology), phospho-pT^308^-Akt (Cat. No. 4056, Cell Signaling), Akt1 (Cat. No. 2938, Cell Signaling), Akt2 (Cat. No. 2964, Cell Signaling), Akt3 (Cat. No. 3788, Cell Signaling), and tubulin α (Cat. No. CP06, Calbiochem). To detect phospho-β-catenin, endogenous β-catenin was immunoprecipitated from cell lysates derived from control and KD_2/3 (A)_ cells using antibodies to β-catenin and subjected to immunoblot analysis with antibodies to phospho-β-catenin (Cat. No. 9561, Cell Signaling). Ectopically expressed Nr2f1 was detected using antibodies to the Flag epitope (Cat. No. 8146, Cell Signaling).

### Tumor and metastasis formation

Orthotopic transplants, intravenous injections (100,000 cells/animal), and scoring of tumor growth and lung metastasis were done as indicated [26, 53]. Animal work was done according to protocols approved by the Bioethics committees of the CSIC, University of Salamanca, and University of Extremadura.

### β-catenin stability

Cells were treated with 50 μM MG132 (Cat No. 474790, Calbiochem) for the indicated periods of time and harvested for immunoblot analyses as indicated [26].

### Genome-wide mRNA and microRNA expression profiling

The Vav2;Vav3-dependent coding transcriptome of 4T1 cells was determined using Affymetrix microarrays (GEO reference, GSE33348) [26]. To characterize the Vav2;Vav3-dependent microRNAome, triplicate samples of total microRNA from control, KD_2/3 (A)_, KD_2/3 (B)_, KD_2/3_+V_2_, KD_2/3_+V_3_, and KD_2/3_+V_2/3_ (Table S1) were analyzed using the GeneChip miRNA 1.0 array (Affymetrix). Data from these arrays have been deposited in the GEO database (GSE97385).

### Bioinformatics of mouse array datasets

Statistical analyses and text processing were carried out using the R (version 3.3.1) and Perl software, respectively. Signal intensity values were obtained from CEL files after robust multichip average [56, 57]. Differentially expressed genes were identified using linear models for microarray data [58]. In this case, adjusted *P* values for multiple comparisons were calculated applying the Benjamini-Hochberg correction method [59]. The *heatmap3* package (http://CRAN.R-project.org/package=heatmap3) was then used to generate the expression heatmaps for the indicated genes. GSEA was performed with the described gene sets using gene set permutations (*n* = 1000) for the assessment of significance and signal-to-noise metric for ranking genes [60]. THE EMT-associated gene signature was obtained from the Molecular Signatures Database (MSigDB) [30]. The Gene Expression Omnibus (GEO) accession codes for other datasets used in this work include GSE25332 (endometrial cancer cell line) [32], GSE40059 (breast cancer cells) [33, 34], and GSE19631 (4TO7 breast cancer cells) [35]. For the discovery of transcription factor binding motifs in the promoters of the deregulated miRNAs, the iRegulon software was used [61]. A collection of 9713 position weight matrices (PWMs) was applied to analyze 10 kb centered around the transcription start site. Motif detection, track discovery, motif-to-factor mapping and target detection were performed with a maximum false discovery rate (FDR) on motif similarity below 0.001. The gene set containing the targets for Nr2f1 was obtained from a previous work [38].

### RNA detection

In the case of microRNAs, total RNA from the cell lines was extracted and purified using the MiRvana kit (Ambion) and quantified by qRT-PCR using the miScript System (Qiagen) following the manufacturer’s recommendations and the iCycler iQ Optical System (BioRad). mRNAs were quantitated as indicated [26]. Primers are available upon request.

### Bioinformatics of human array datasets from tumors and breast cancer cell lines

Heatmaps were plotted using the *heatmap3* package, as indicated above. Coexpression matrices were calculated from the corresponding expression matrices for the indicated genes using the *corrplot* package (http://CRAN.R-project.org/package=corrplot). Correlations with a statistically significant (*P* value < 0.05) Pearson correlation coefficient (*r*) according to the number of samples were labeled with asterisks in the figures. These statistically significant values were achieved with *r* > 0.23 (in the case of dataset 1), > 0.13 (in the case of dataset 2), and > 0.17 (in the case of dataset 3). Survival analyses were performed through Kaplan-Meier estimates [62] of distant recurrence-free survival according to the level of enrichment of the described transcriptional signatures. This enrichment was calculated using ssGSEA [28, 60, 63] and, subsequently, the Mantel-Cox test [64] was applied to statistically validate the differences between the survival distributions. The GEO accession codes for the datasets used were GSE36133 (for the Cancer Cell Line Encyclopedia expression analyses) [65], GSE65194 (dataset 1) [66], GSE25066 (dataset 2) [67, 68] and GSE78958 (dataset 3) [69] (for the coexpression analyses), GSE25066 [67, 68] and GSE2603 [70] (for the survival analyses), and GSE2603 [70] (metastasis free-survival).

### Statistics

For most of the wet lab data presented in this work, Shapiro-Wilk normality tests were applied before the final Student’s *t*-tests. In other cases, (e.g., Fig. 4d,e), data were analyzed using Mann-Whitney tests. Sample size and number of independent experiments for each experiment is indicated in the appropriate figure legend. Experimental values in graphs are provided as mean and s.e.m. Results with *P* values ≤ 0.05 were considered statistically significant.

## Electronic supplementary material

Supplemental Information
