# Supplementary material for: Vav proteins maintain epithelial traits in breast cancer cells using miR-200c-dependent and independent mechanisms
Source: Oncogene. 2018 Aug 7;38(2):209–27. doi: 10.1038/s41388-018-0433-7 (PMC6230471; doi:10.1038/s41388-018-0433-7)
Supplement: Supplementary file 1 — Supplemental Information [file 41388_2018_433_MOESM1_ESM.pdf]

Supplemental Information for

Vav proteins maintain epithelial traits in breast cancer cells using *miR-200c*-dependent and independent mechanisms

by

L. Francisco Lorenzo-Martín, Carmen Citterio, Mauricio Menacho-Márquez, Javier Conde, Romain M. Larive, Sonia Rodríguez-Fdez, Ramón García-Escudero, Javier Robles-Valero, Myriam Cuadrado, Isabel Fernández-Pisonero, Mercedes Dosil, María A. Sevilla, María J. Montero, Pedro M. Fernández-Salguero, Jesús M. Paramio, and Xosé R. Bustelo*

*To whom correspondence should be addressed. E-mail: xbustelo@usal.es

This PDF file includes:

(1) Supplemental Figures S1 to S6 and legends (pages 2 to 8)

(2) Supplemental Tables S1 (pages 9 and 11) and S2 (pages 12 and 14)

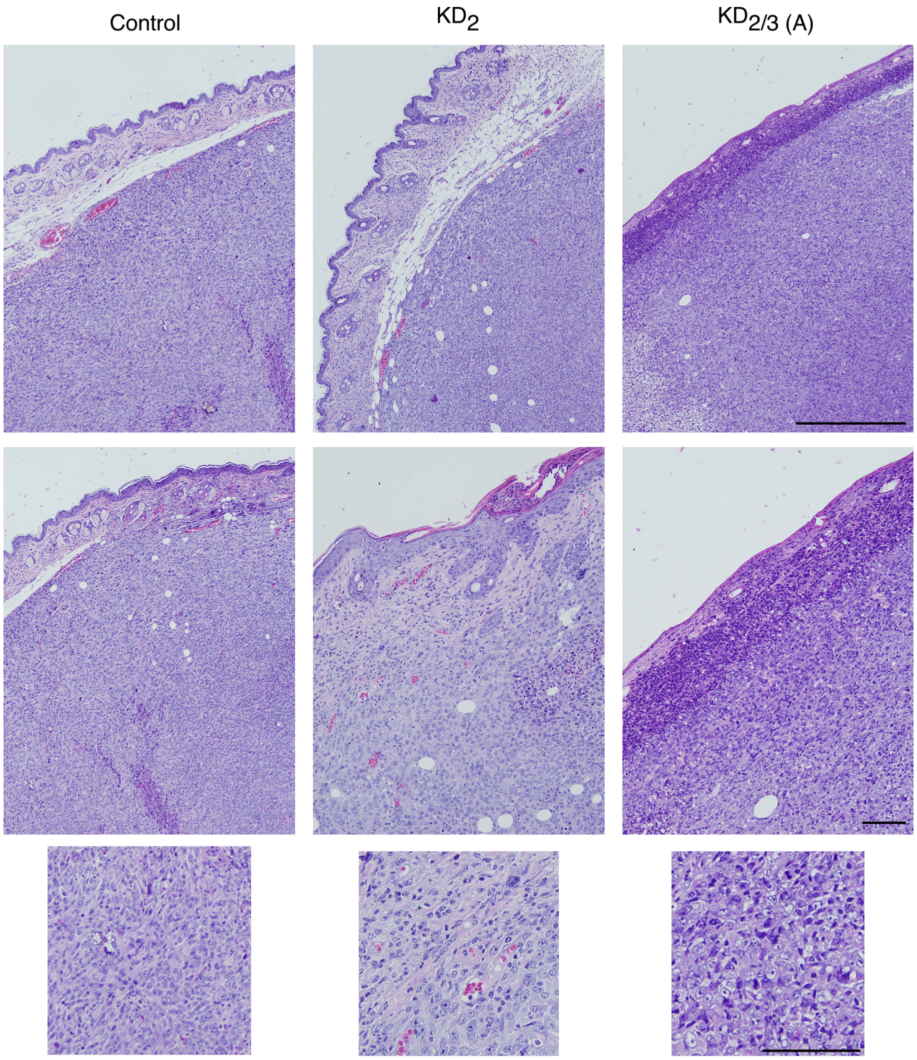

FIGURE S1. Vav2 and Vav3 are required to maintain epithelial traits in breast cancer cells. Examples of the histology of tumors derived from the indicated xenografted 4T1 cells. Scale bar, 500 (top panels) and 100 (middle panels) µm. The bottom panels show 300 x zooms of areas within the middle panels that were made with the software used for figure generation (Canvas Draw 2 for Mac, ACD Systems).

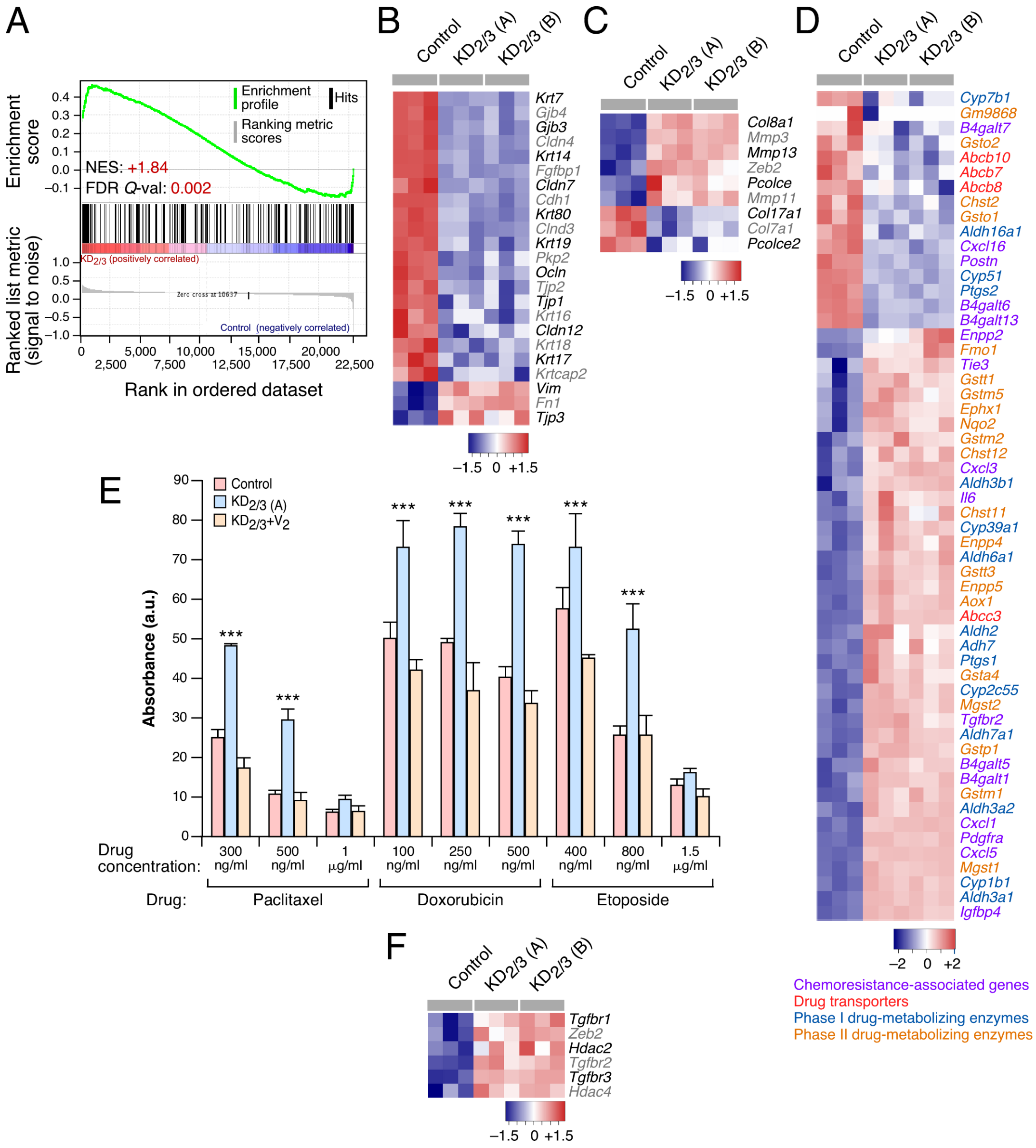

FIGURE S2. The Vav coding transcriptome and microRNAome are linked to EMT programs. (A) GSEA showing the overlap of the Vav-regulated 4T1 cell transcriptome with a previously described EMT-associated transcriptome present in MSigDB (see Materials and Methods). The normalized enrichment scores (NES) and false discovery rate values (FDR, using *q* values) are indicated inside each GSEA graph. *Q*-val, q value. **(B-D)** Heatmap showing epithelial state- (B), mesenchymal state- (C), and chemoresistance-associated (D) transcripts that are up- (red) and downregulated (blue) in indicated 4T1 cell lines (top). Triplicates for each cell line (columns) are shown. Relative changes in abundance are shown in color gradients according to the scale shown at the bottom. In C, genes are colored according to their role in chemoresistance regulation (bottom). **(E)** Response of 4T1 cell derivatives (inset) to the indicated concentrations of chemotherapy agents (bottom). Values represent the variation relative to untreated cells (which was given an arbitrary value of 100). ***, $P \leq 0.001$ ($n = 3$). **(F)** Heatmap of the transcripts encoding EMT regulators that show statistically significant variations between control and two independent clones of $KD_{2/3}$ cells. 32 additional EMT regulatory factors compiled in previous reviews[11, 26] that show no statistically significant changes in these analyses are not shown. Changes in abundance are plotted as in B.

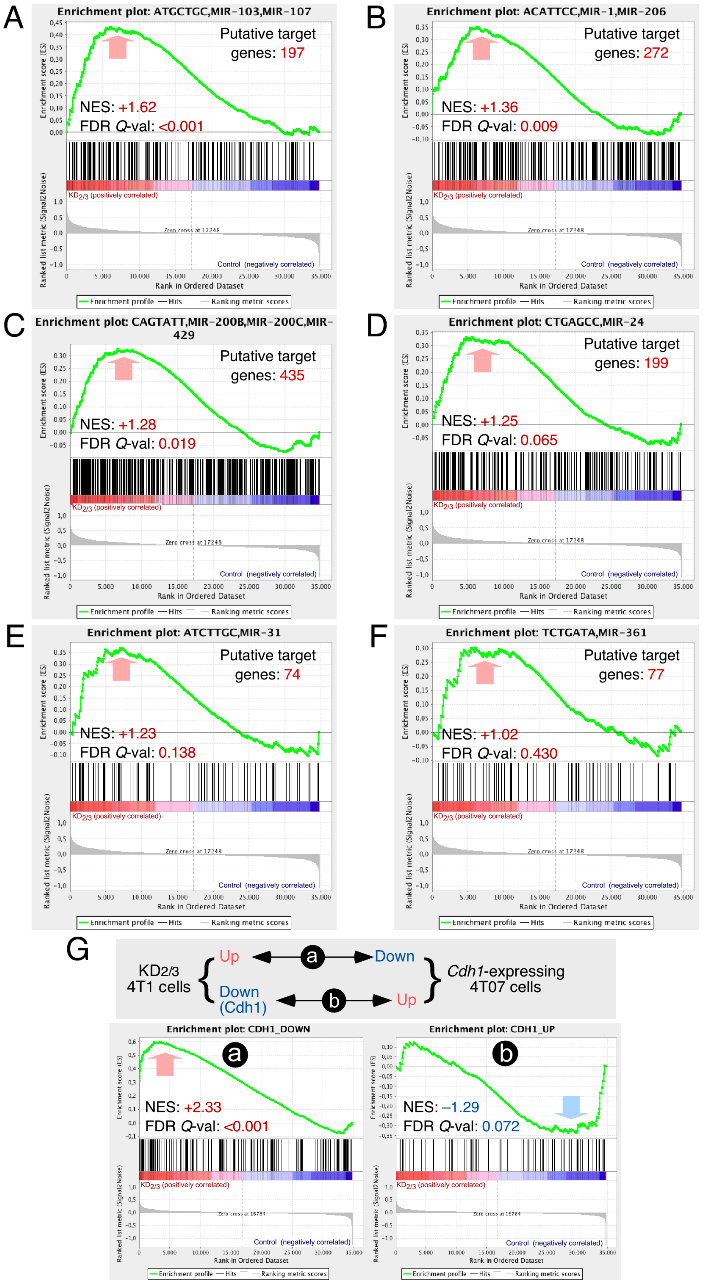

FIGURE S3. Characterization of the Vav-dependent microRNAome. (A-F) GSEA showing the percentage of the Vav2;Vav3-dependent coding transcriptome that contains target sequences for *miR-103* (A), *miR-107* (A), *miR-206* (B), *miR-200* family (C), *miR-24* (D), *miR-31* (E), and *miR-361* (F). The NES and FDR values are shown inside each graph as indicated in **Fig. S2A**. *miR-361* does no reach statistical significance in these analyses. The number of putative transcript targets for each microRNA is indicated in the top right of each panel. Other Vav-regulated microRNAs were not included due to lack of enough information about its target mRNA sequences. **(G)** Top panel, scheme of the similarities expected according to the mechanistic model proposed in this study. Bottom panels, GSEAs showing the similarities of the transcriptomal subsets indicated in top. The NES and FDR values are shown inside each graph as indicated in **Fig. 3B**. Red and blue values represent positive and negative correlation between the compared transcriptomes.

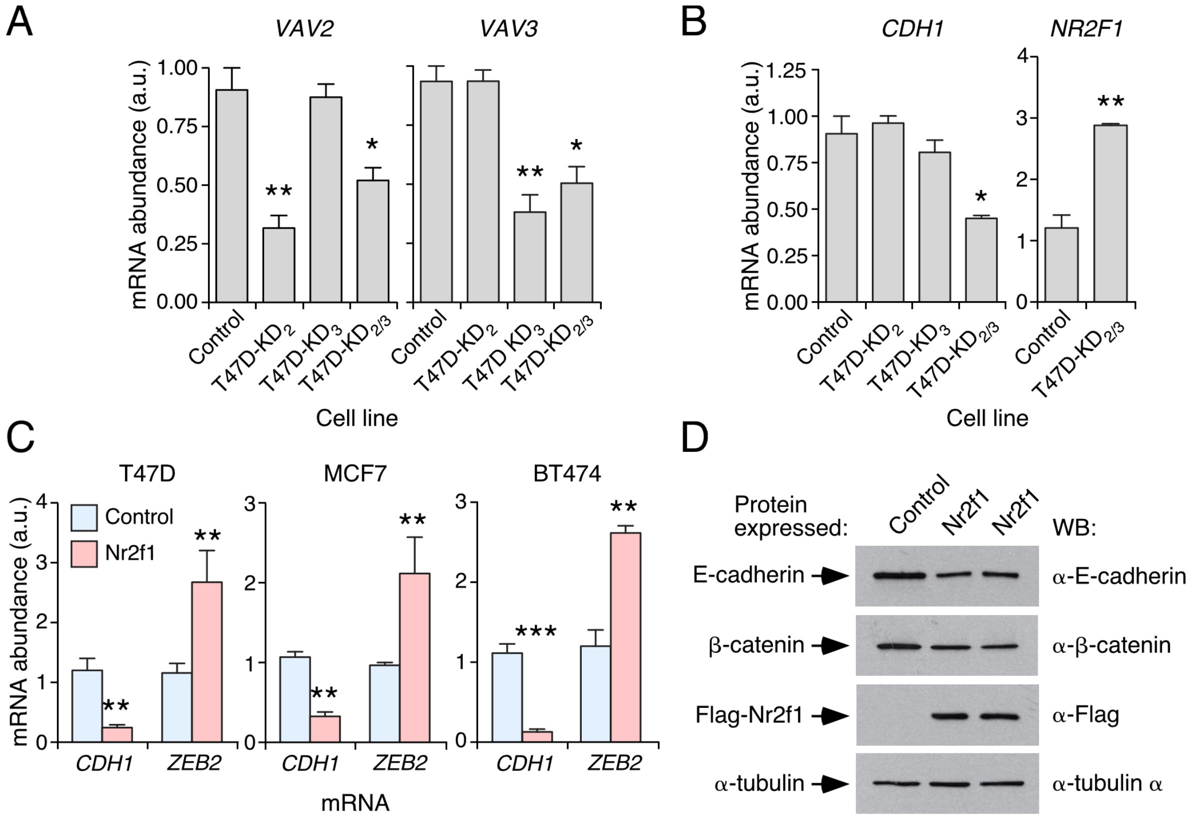

FIGURE S4. Vav and Nr2f1 proteins negatively regulate E-cadherin in human breast cancer cells. (A) Abundance of *VAV2* (left panel) and *VAV3* (right panel) mRNAs in control and T47D cell derivatives. *, $P \leq 0.05$; **, $P \leq 0.01$ ($n = 3$). **(B)** Abundance of *CDH1* (left) and *NR2F1* (right) transcripts in indicated T47D cells. *, $P \leq 0.05$; **, $P \leq 0.01$ ($n = 3$). **(C)** Abundance of indicated transcripts (bottom) upon the transient expression of Nr2f1 in indicated cell lines (top). **, $P \leq 0.01$; ***, $P \leq 0.001$ relative to the appropriate control (which was given an arbitrary value of 1) ($n = 3$). **(D)** Expression of indicated proteins (left) in control and two independently sets of T47D cells transiently transfected with Nr2f1. As loading control, we used the abundance of endogenous tubulin α (bottom panel) ($n = 2$).

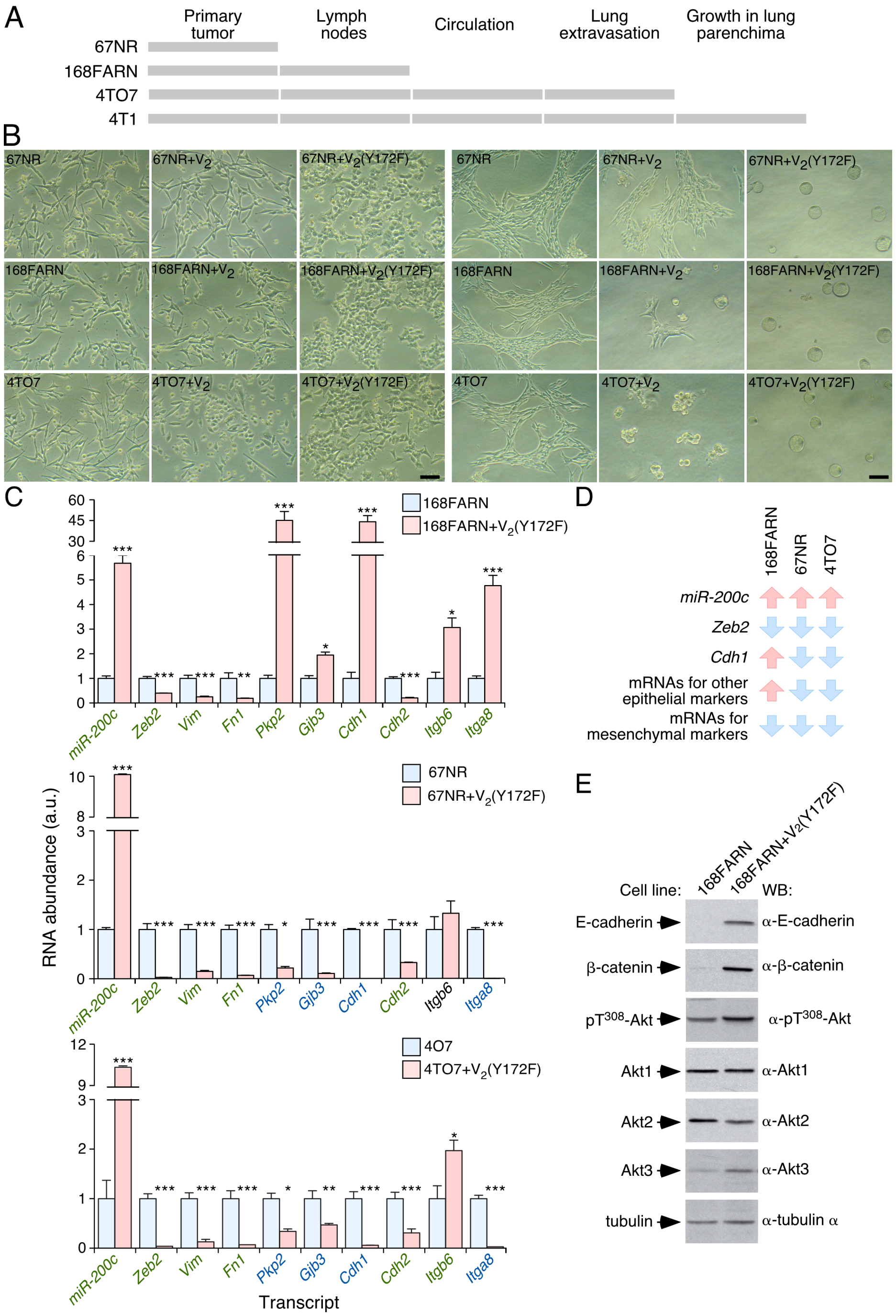

FIGURE S5. Constitutively active Vav2 triggers MET in mesenchymal breast cancer cells using E-cadherin dependent and independent mechanisms. **(A)** Schematic representation of the metastatic defects exhibited by indicated breast cancer cells. Bars depict the metastatic stage in which those cell lines are proficient. **(B)** Representative examples of the morphology of indicated cell lines in 2D (left panels) and 3D (right panels) cultures. Scale bars, 50 µm. **(C)** Abundance of *miR-200c* and indicated coding transcripts in 168 FARN (top), 67NR (middle) and 4TO7 (bottom) cells that were transduced with control (light blue) and Vav2^{Y172F}-encoding (light red) lentiviral particles (inset). V2(Y172F), Vav2^{Y172F}. Transcripts with an expected regulation according to the pathway dissected in 4T1 cells are highlighted in green font. Those that show an

unexpected pattern of expression are highlighted in blue font. *, $P \leq 0.05$; **, $P \leq 0.01$; ***, $P \leq 0.001$ (n = 3). **(D)** Summary of the results shown obtained in panel C. The cell lines and transcripts are indicated at the top and the left, respectively. Upregulation and downregulation events are depicted as red and blue arrows, respectively. **(E)** Expression of indicated proteins and phospho-proteins (left) in control and Vav2^{Y172F}-expressing 168FARN cells. As loading control, we used the abundance of endogenous Akt (fourth panel from top) and tubulin α (bottom panel) (n = 2).

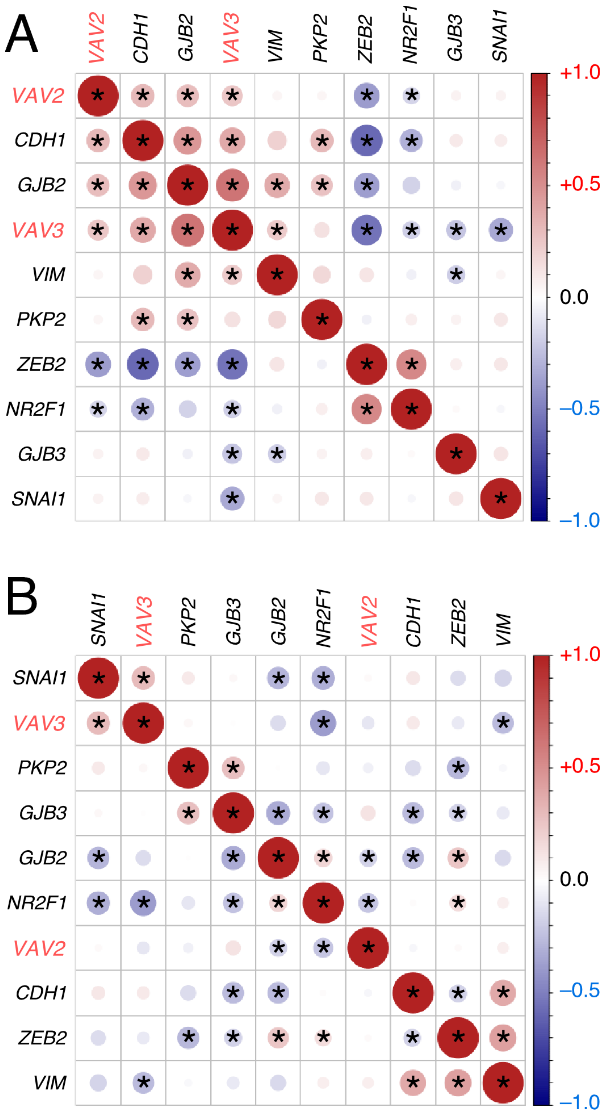

FIGURE S6. The Vav2-dependent anti-EMT and prometastatic program in human tumors. (A,B) Expression correlation matrix of indicated transcripts in human luminal (A) and basal (B) breast tumor samples from the GSE65194 microarray dataset. Values are plotted as in **Fig. 7A**.

TABLE S1. Cell lines used in this study (it continues in the next page)

Cell line	Features
Control	4T1 cells harboring the empty pLKO.1puro lentiviral vector
KD_2	A clone (A) of *Vav2*-deficient 4T1 cells generated by transducing an shRNA-encoding lentivirus
KD_3	A clone (A) of *Vav3*-deficient 4T1 cells generated by transducing an shRNA-encoding lentivirus
$KD_{2/3\ (A)}$	A clone (A) of double *Vav2;Vav3*-deficient 4T1 cells generated by transducing lentiviral particles each encoding shRNAs to mouse *Vav2* and *Vav3* transcripts
$KD_{2/3\ (B)}$	A clone (B) of double *Vav2;Vav3*-deficient 4T1 cells generated by transducing lentiviral particles each encoding shRNAs to mouse *Vav2* and *Vav3* transcripts
$KD_{2/3+ev}$	$KD_{2/3\ (A)}$ cells harboring an empty pLVX-IRES-Hyg lentiviral vector
$KD_{2/3}+V_2$	$KD_{2/3\ (A)}$ cells in which an HA-tagged version of wild type Vav2 was re-expressed using lentiviral delivery methods
$KD_{2/3}+V_{2(R373A)}$	$KD_{2/3\ (A)}$ cells in which an HA-tagged, catalytically inactive version of Vav2 was re-expressed using lentiviral delivery methods
$KD_{2/3}+V_3$	$KD_{2/3\ (A)}$ cells in which an Myc-tagged version of wild type Vav3 was re-expressed using lentiviral delivery methods
$KD_{2/3}+V_{2/3}$	$KD_{2/3\ (A)}$ cells in which the wild type versions of Vav2 (HA-tagged) and Vav3 (Myc-tagged) were re-expressed using lentiviral delivery methods
$KD_{2/3}$+200c	$KD_{2/3\ (A)}$ cells overexpressing miR-200c plus GFP (introduced using lentiviral delivery methods)
$KD_{2/3}+Rac1^{Q61L}$	$KD_{2/3\ (A)}$ cells in which a constitutively active version of Rac1 was stably expressed using lentiviral delivery methods

TABLE S1. Cell lines used in this study (it continues from previous page)

Cell line	Features
$KD_{2/3}$+Pak1^{T423E}	$KD_{2/3\ (A)}$ cells in which a constitutively active version of Pak1 was stably expressed using lentiviral delivery methods
$KD_{2/3}$+PI3K^{CAAX}	$KD_{2/3\ (A)}$ cells in which a constitutively active version of PI3K α (carrying the CAAX box of K-RAS) was stably expressed using lentiviral delivery methods
KD-Raptor	A pool of *Rptor*-deficient 4T1 cells generated by transducing an shRNA-encoding lentivirus
T47D-KD_2	A pool of *VAV2*-deficient T47D cells generated by transducing an shRNA-encoding lentivirus
T47D-KD_3	A pool of *VAV3*-deficient T47D cells generated by transducing an shRNA-encoding lentivirus
T47D-$KD_{2/3}$	A pool of *VAV2;VAV3*-deficient T47D cells generated by transducing an shRNA-encoding lentivirus encoding shRNAs to human *VAV2* and *VAV3* transcripts
KD-Itgb6	A clone (A) of integrin β6-deficient 4T1 cells generated by transducing an shRNA-encoding lentivirus
KD-Itga8	A clone (A) of integrin α8-deficient 4T1 cells generated by transducing a shRNA-encoding lentivirus
KD-Ilk	A clone (A) of Ilk-deficient 4T1 cells generated by transducing a shRNA-encoding lentivirus
KD-Tacstd2	A clone of Tacstd2-deficient 4T1 cells generated by transducing an shRNA-encoding lentivirus
KD-Inhba	A clone of Inhibin βA-deficient 4T1 cells generated by transducing an shRNA-encoding lentivirus
KD-Ptgs2	A clone of cyclo-oxigenase 2-deficient 4T1 cells generated by transducing an shRNA-encoding lentivirus

TABLE S1. Cell lines used in this study (it continues from previous pages)

Cell line	Features
67NR+V_2	67NR cells in which the wild type version of Vav2 (HA-tagged) was overexpressed using lentiviral delivery methods
67NR+$V_{2(Y172F)}$	67NR cells in which a constitutively active version of Vav2 (HA-tagged) was overexpressed using lentiviral delivery methods
4OT7+V_2	4OT7 cells in which the wild type version of Vav2 (HA-tagged) was overexpressed using lentiviral delivery methods
4OT7+$V_{2(Y172F)}$	4OT7 cells in which a constitutively active version of Vav2 (HA-tagged) was overexpressed using lentiviral delivery methods
168FARN+V_2	168FARN cells in which the wild type version of Vav2 (HA-tagged) was overexpressed using lentiviral delivery methods
168FARN+$V_{2(Y172F)}$	168FARN cells in which a constitutively active version of Vav2 (HA-tagged) was overexpressed using lentiviral delivery methods

TABLE S2. List of genes included in the *miR-200c*–Vav-dependent EMT signature (it continues in next pages)

Probeset	Gene symbol	Gene description
10355984	*Serpine2*	Serine (or cysteine) peptidase inhibitor clade E member 2
10532085	*Tgfbr3*	Transforming growth factor β receptor III
10546450	*Adamts9*	A disintegrin-like and metallopeptidase with thrombospondin type 1 motif 9
10546432	*Adamts9*	A disintegrin-like and metallopeptidase with thrombospondin type 1 motif 9
10522503	*Pdgfra*	Platelet derived growth factor receptor, α polypeptide
10594800	*Fam63b*	Family with sequence similarity 63 member B
10422321	*Dzip1*	DAZ interacting protein 1
10546454	*Adamts9*	A disintegrin-like and metallopeptidase (reprolysin type) with thrombospondin type 1 motif 9
10435271	*Heg1*	HEG homolog 1
10606186	*Slc16a2*	Solute carrier family 16 member 2
10546434	*Adamts9*	A disintegrin-like and metallopeptidase with thrombospondin type 1 motif 9
10429160	*St3gal1*	ST3 β-galactoside α-2,3-sialyltransferase 1
10504424	*Reck*	Reversion-inducing-cysteine-rich protein with kazal motifs
10435266	*Heg1*	HEG homolog 1
10585982	*Myo9a*	Myosin ixa
10397966	*Otub2*	OTU domain, ubiquitin aldehyde binding 2
10585970	*Myo9a*	Myosin IXa
10585956	*Myo9a*	Myosin IXa
10521759	*Slit2*	Slit homolog 2
10545974	*Antxr1*	Anthrax toxin receptor 1
10585988	*Myo9a*	Myosin IXa
10360764	*Enah*	Enabled homolog
10585972	*Myo9a*	Myosin IXa
10398240	*Eml1*	Echinoderm microtubule associated protein like 1
10591816	*Dpy19l1*	Dpy-19-like
10523297	*Ccng2*	Cyclin G2
10574166	*Cpne2*	Copine II
10585986	*Myo9a*	Myosin IXa
10457508	*Npc1*	Niemann Pick type C1
10403727	*Gli3*	GLI-Kruppel family member GLI3
10568785	*Bnip3*	BCL2/adenovirus E1B interacting protein 3
10585984	*Myo9a*	Myosin IXa
10585980	*Myo9a*	Myosin IXa
10585978	*Myo9a*	Myosin IXa
10585976	*Myo9a*	Myosin IXa
10544885	*Fkbp14*	FK506 binding protein 14
10390574	*Fbxl20*	F-box and leucine-rich repeat protein 20
10446334	*Glcci1*	Glucocorticoid induced transcript 1
10439798	*Dzip3*	DAZ interacting protein 3
10506031	*Nfia*	Nuclear factor I/A
10585974	*Myo9a*	Myosin IXa
10478219	*Plcg1*	Phospholipase C-γ1
10586039	*Tle3*	Transducin-like enhancer of split 3
10426611	*Cacnb3*	Calcium channel, voltage-dependent β3 subunit

TABLE S2. List of genes included in the *miR-200c*–Vav-dependent EMT signature (it continues from page 12)

Probeset	Gene symbol	Gene description
10542740	*Sspn*	Sarcospan
10376208	*Gm2a*	GM2 ganglioside activator protein
10370210	*Col6a1*	Collagen type VI α1
10414269	*Bnip3*	BCL2/adenovirus E1B interacting protein 3
10356339	*Pde6d*	Phosphodiesterase 6D δ
10462281	*Vldlr*	Very low density lipoprotein receptor
10395103	*Pxdn*	Peroxidasin homolog
10376950	*Pmp22*	Peripheral myelin protein 22
10594802	*Fam63b*	Family with sequence similarity 63B
10487392	*Kcnip3*	Kv channel interacting protein 3
10585992	*Myo9a*	Myosin IXa
10585990	*Myo9a*	Myosin IXa
10584941	*Bace1*	Beta-site APP cleaving enzyme 1
10524436	*Usp30*	Ubiquitin specific peptidase 30
10527101	*Foxk1*	Forkhead box K1
10546430	*Adamts9*	A disintegrin-like and metallopeptidase with thrombospondin type 1 motif 9
10483698	*Wipf1*	WAS/WASL interacting protein family, member 1
10420823	*Hmbox1*	Homeobox containing 1
10592734	*Cbl*	Casitas B-lineage lymphoma
10531796	*Wdfy3*	WD repeat and FYVE domain containing 3
10452613	*Arhgap28*	Rho GTPase activating protein 28
10537353	*Ttc26*	Tetratricopeptide repeat domain 26
10483865	*Fkbp7*	FK506 binding protein 7
10592719	*Oaf*	OAF homolog
10458843	*Sema6a*	Semaphorin 6A
10451613	*Foxp4*	Forkhead box P4
10516427	*Ncdn*	Neurochondrin
10593384	*Dixdc1*	DIX domain containing 1
10378988	*Phf12*	PHD finger protein 12
10542738	*Rassf8*	Ras association family member 8
10358816	*Lamc1*	Laminin γ 1
10547597	*Phc1*	Polyhomeotic-like 1
10549361	*Tm7sf3*	Transmembrane 7 superfamily member 3
10440849	*Synj1*	Synaptojanin 1
10572786	*Ap1m1*	Adaptor-related protein complex AP-1 µ1
10535883	*Katnal1*	Katanin p60 subunit A-like 1
10426798	*Smarcd1*	SWI/SNF related, matrix associated, actin dependent regulator of chromatin D1
10390022	*Rsad1*	Radical S-adenosyl methionine domain containing 1
10575120	*Sntb2*	Syntrophin basic 2
10582874	*Sp110*	Sp110 nuclear body protein
10496387	*Dnajb14*	Dnaj (Hsp40) homolog B14
10380477	*Ppp1r9b*	Protein phosphatase 1 regulatory subunit 9B

TABLE S2. List of genes included in the *miR-200c*–Vav-dependent EMT signature (it continues from pages 12 and 13)

Probeset	Gene symbol	Gene description
10370510	*Syde1*	Synapse defective 1 Rho GTPase homolog 1
10586458	*Csnk1g1*	Casein kinase 1-γ1
10376690	*Llgl1*	Lethal giant larvae homolog 1
10486595	*Ttbk2*	Tau tubulin kinase 2
10401637	*Nek9*	NIMA (never in mitosis gene α)-related expressed kinase 9
10436372	*Dcbld2*	Discoidin, CUB and LCCL domain containing 2
10375103	*Fbxw11*	F-box and WD-40 domain protein 11
10546452	*Adamts9*	A disintegrin-like and metallopeptidase with thrombospondin type 1 motif 9
10345715	*Map4k4*	Mitogen-activated protein kinase kinase kinase kinase 4
10400515	*Sec23a*	Sec23a
10495896	*Camk2d*	Calcium/calmodulin-dependent protein kinase II δ
10468992	*Frmd4a*	FERM domain containing 4A
10361055	*Vash2*	Vasohibin 2
10444268	*Tap2*	Transporter 2 ATP-binding cassette sub-family B
10428857	*Mtss1*	Metastasis suppressor 1
10496324	*Slc39a8*	Solute carrier family 39 member 8
10353272	*Stau2*	Staufen homolog 2
10417095	*Farp1*	FERM, RhoGEF and pleckstrin domain protein 1
10469475	*Mllt10*	Myeloid/lymphoid or mixed-lineage leukemia 10
10505030	*Fsd1l*	Fibronectin type III and SPRY domain containing 1-like
10511446	*Asph*	Aspartate-β-hydroxylase
10569134	*Deaf1*	Deformed epidermal autoregulatory factor 1
10527012	*Lfng*	LFNG O-fucosylpeptide 3-β-N-acetylglucosaminyltransferase
10425354	*Mgat3*	Mannoside acetylglucosaminyltransferase 3
